# Mitochondrial dysfunction enhances influenza pathogenesis by up-regulating de novo sialic acid biosynthesis

**DOI:** 10.1126/sciadv.adu3739

**Published:** 2025-07-04

**Authors:** Amanda L. Fuchs, Bharati Singh, Jillian W. Jetmore, Emily B. Warren, Payal Banerjee, Jose L. Marin Franco, Michael A. Eckhaus, Tatiana N. Tarasenko, Madeleine R. Assaad, Ivan Kosik, Jonathan Yewdell, Peter J. McGuire

**Affiliations:** ^1^ Metabolism, Infection, and Immunity Section, Metabolic Medicine Branch, National Human Genome Research Institute, National Institutes of Health, Bethesda, MD, USA.; ^2^ Division of Veterinary Resources, Office of Research Services, National Institutes of Health, Bethesda, MD, USA.; ^3^ Cellular Biology Section, National Institute of Allergy and Infectious, National Institutes of Health, Bethesda, MD, USA.

## Abstract

Mitochondrial dysfunction can trigger metabolic adaptations that resemble those induced by influenza A virus (IAV) infection. Here, we show that oxidative phosphorylation (OXPHOS) impairment, modeled by *Ndufs4* deficiency, reprograms lung epithelial metabolism to promote IAV pathogenesis. In both *Ndufs4* knockout (KO) mice and lung epithelial cells, OXPHOS deficiency increased glycolytic flux, diverting carbons into hexosamine and de novo sialic acid (SIA) biosynthesis pathways. This led to elevated sialylation and enhanced viral attachment. In *Ndufs4* KO models, adenosine monophosphate–activated protein kinase signaling was insufficient to blunt this increased metabolic flux. IAV infection further exacerbated this metabolic vulnerability, amplifying SIA and viral burden. Pharmacologic rerouting of glucose carbons with dichloroacetate reduced sialylation, viral replication, and inflammatory responses in *Ndufs4* KO models. These findings reveal that mitochondrial dysfunction enhances IAV susceptibility by disrupting energy sensing and fueling viral receptor biosynthesis, highlighting the importance of epithelial metabolism in viral pathogenesis and suggesting metabolic modulation as a potential therapeutic.

## Teaser

Mitochondrial dysfunction enhances glycolysis-driven sialylation, increasing influenza receptor density and infectious burden.

## INTRODUCTION

Under normal conditions, the lung relies on oxidative phosphorylation (OXPHOS) as the primary pathway for generating adenosine triphosphate (ATP), which is necessary for maintaining critical functions such as secretion, ion transport, and cellular maintenance (*1*). Although glucose is the main fuel source for OXPHOS, fatty acids and amino acids can also contribute to ATP production when needed. Environmental and pathological stressors can lead to mitochondrial dysfunction with a depression of OXPHOS in lung respiratory epithelial cells (*2*, *3*). When OXPHOS is impaired, cells may resort to alternative metabolic pathways to sustain energy production. This adaptive process, termed metabolic reprogramming or metabolic allostasis, typically includes a shift to aerobic glycolysis and the activation of anabolic pathways (*4*).

The metabolic shift seen in mitochondrial dysfunction bears notable similarities to the metabolic adaptations observed in respiratory epithelial cells during influenza A virus (IAV) infection (*5*–*7*). The viral life cycle imposes high metabolic demands on cells, leading to extensive reprogramming to support viral replication. This shared metabolic phenotype suggests that individuals with secondary mitochondrial dysfunction, such as those with chronic respiratory diseases (CRDs) like asthma or chronic obstructive pulmonary disease (*8*), may have a cellular environment that is more conducive to IAV infection. CRDs face increased vulnerability to IAV infection, which further elevates morbidity and mortality rates (*9*).

Supporting the idea of mitochondrial dysfunction enhancing viral pathogenesis, studies have shown that children with mitochondrial diseases (MtDs) experience more severe or prolonged respiratory viral infections (*10*). On the basis of the above findings, we hypothesized that mitochondrial dysfunction exacerbates IAV pathogenesis by creating a metabolic environment that enhances viral pathogenesis and intensifies disease severity. To help define the mechanistic underpinnings linking mitochondrial dysfunction and IAV pathogenesis, we used models of OXPHOS deficiency due to an impairment in NADH dehydrogenase (complex I): *Ndufs4* knockout (KO) mice and mouse lung epithelial (LET1) cells (*11*). Our findings indicate that metabolic allostatic mechanisms in respiratory epithelial cells with mitochondrial dysfunction contribute to heightened susceptibility to influenza infection. This increased vulnerability is due to enhanced expression of sialic acid (SIA), the surface receptor for influenza. Our study highlights the pivotal role of metabolic allostasis due to mitochondrial dysfunction in influenza pathogenesis.

## RESULTS

### *Ndufs4* KO mice demonstrate enhanced morbidity with IAV infection

**Fig. 1.**
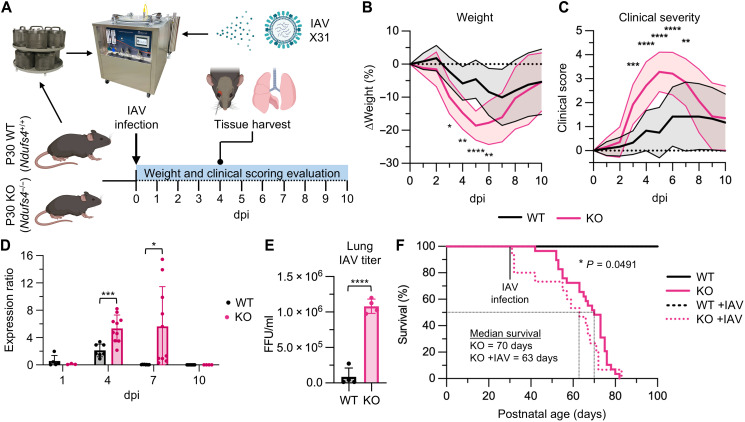
*Ndufs4* KO mice demonstrate enhanced morbidity with IAV infection. (**A**) IAV infection schematic. (**B**) Body weight and (**C**) clinical scoring data during IAV infection. Data were analyzed using an ordinary two-way analysis of variance (ANOVA) with Šídák’s multiple comparisons test, *n* = 12 for WT and *n* ≥ 13 for KO. (**D**) Lung IAV *NS1* at 1, 4, 7, and 10 dpi, *n* ≥ 5 for WT and *n* ≥ 3 for KO. (**E**) Lung IAV titer FFU/ml data at 4 dpi, *n* = 4 for WT and KO. (**F**) Kaplan-Meier survival curve data. Data were analyzed using a Gehan-Breslow-Wilcoxon test, *n* = 28 for WT and WT +IAV, *n* = 29 for KO, and *n* = 15 for KO +IAV. Data in (B) to (F) are presented as means ± SD and were analyzed using unpaired *t* tests with Welch’s correction, unless otherwise indicated.

Viral infections in children with MtDs tend to be more severe and have a prolonged course, suggesting that mitochondrial dysfunction may play a role in enhancing pathogenesis (*10*). To better understand how mitochondrial dysfunction influences viral pathogenesis, we infected *Ndufs4* KO and wild-type (WT) mice with mouse-adapted IAV, X31, at postnatal age 30 (P30) using a whole-body inhalation exposure system to produce viral pneumonia (Fig. 1A). Body weight, a reliable biomarker of illness in mice during IAV infection (*12*–*14*), was reduced in *Ndufs4* KO mice at 3 to 6 days postinfection (dpi) relative to WT mice (Fig. 1B). Correspondingly, clinical severity scoring (CSS; criteria described in table S1) was elevated in *Ndufs4* KO mice at 3 to 7 dpi relative to WT mice (Fig. 1C). On the basis of this increased morbidity, we next examined lung viral load. Real-time reverse transcription polymerase chain reaction (RT-PCR) of IAV nonstructural protein 1, *NS1,* indicated increased viral load at 4 dpi in *Ndufs4* KO lungs relative to WT (Fig. 1D). For some *Ndufs4* KO mice, this viral load remained elevated at 7 dpi, with eventual resolution in all animals by 10 dpi, which coincided with an improvement in weight and clinical scores (Fig. 1, B and C). Correspondingly, lung IAV titers at 4 dpi were also elevated in *Ndufs4* KO mice (Fig. 1E). Because we observed a higher viral burden in *Ndufs4* KO lungs, we next assessed histological and pathological indicators of lung injury. Histological evaluation and pathology scoring of *Ndufs4* KO mouse lung tissue at 4 dpi was comparable to WT (fig. S1, A and B). In addition, lactate dehydrogenase (LDH) activity levels in bronchoalveolar lavage (BAL) fluid from *Ndufs4* KO mice at 4 dpi were not distinct from WT (fig. S1C), indicating that IAV-induced lung injury is not exacerbated in *Ndufs4* KO mice relative to WT. These findings are consistent with a prior report showing that, although viral loads may peak early (3 to 4 dpi), severe histological damage typically emerges later (*15*). With IAV infection, *Ndufs4* KO mice demonstrated reduced lifespan relative to their uninfected (UI) counterparts, with median survival being 70 and 63 days for UI *Ndufs4* KO and IAV-infected *Ndufs4* KO mice, respectively (Fig. 1F). Notably, around 20% of *Ndufs4* KO mice died shortly after infection was induced.

### Systemic inflammation is independent of T cell and macrophage *Ndufs4* deficiency

**Fig. 2.**
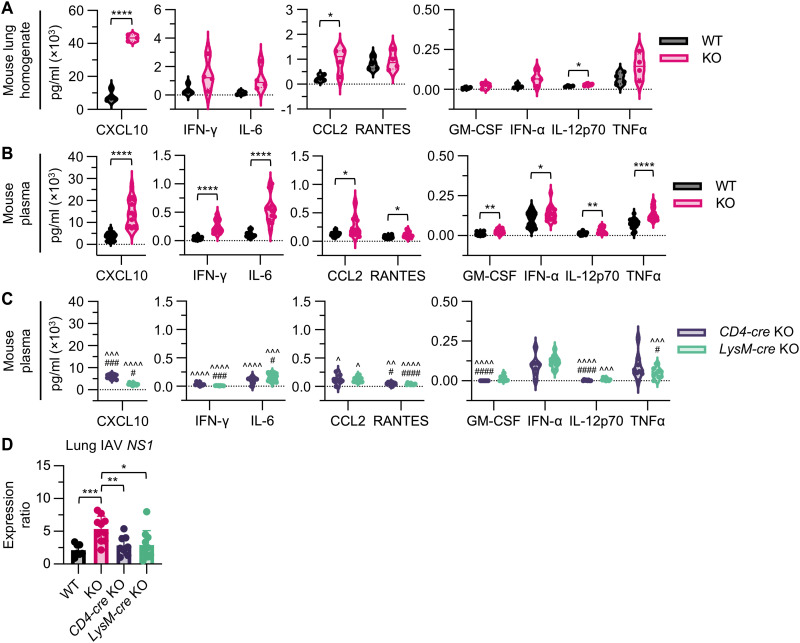
Systemic inflammation is independent of T cell and macrophage *Ndufs4* deficiency. (**A**) Cytokine levels in lung homogenate samples obtained from WT and KO mice at 4 dpi. Data were analyzed using an unpaired *t* test, *n* = 4 for WT and *n* ≥ 3 for KO. (**B**) Cytokine levels in peripheral plasma samples obtained from WT and KO mice at 4 dpi, *n* ≥ 18 for WT and *n* ≥ 16 for KO. (**C**) Cytokine levels in peripheral plasma samples obtained from *CD4-* and *LysM-cre* KO mice at 4 dpi, *n* ≥ 7 for *CD4-cre* KO and *n* ≥ 9 for *LysM-cre* KO. (**D**) Lung IAV *NS1* in WT, KO, *CD4-cre* KO, and *LysM-cre* KO mice at 4 dpi, *n* = 7 for WT, *n* = 10 for KO and *LysM-cre* KO, and *n* = 8 for *CD4-cre* KO. Data in (A) to (D) are presented as means ± SD and were analyzed using unpaired *t* tests with Welch’s correction, unless otherwise indicated.

To better understand whether the marked illness at 4 dpi was associated with altered immune responses, we next examined cytokine profiles in *Ndufs4* KO mice. Lung homogenate samples displayed an elevation of CCL2, CXCL10, and interleukin-12p70 (IL-12p70) in *Ndufs4* KO mice relative to WT (Fig. 2A). Interferons and several inflammatory cytokines also showed increased levels, although the differences were not statistically significant. These findings indicate that *Ndufs4* KO mice display enhanced early phases of the antiviral immune response with elevated recruitment signals for immune cells. We next examined peripheral cytokine levels to assess whether systemic immune activation accompanied the local lung environment response. Systemic hypercytokinemia was observed in *Ndufs4* KO mice as demonstrated by increased plasma levels of CCL2, CXCL10, granulocyte-macrophage colony-stimulating factor (GM-CSF), interferon-α (IFN-α), IFN-γ, IL-6, IL-12p70, RANTES, and tumor necrosis factor–α (TNFα) (Fig. 2B). These findings suggest that systemic hypercytokinemia in *Ndufs4* KO mice may result from dysregulated peripheral immune sensing or amplification, rather than excessive local inflammation in the lungs. *Ndufs4* KO mice have been reported to display heightened inflammation (*16*). To determine whether immune cells were driving this response in *Ndufs4* KO, we assessed two key cell populations in the antiviral response: T cells and macrophages. Quantitation of plasma cytokine levels in IAV-infected *CD4-* and *LysM-cre* conditional *Ndufs4* KO mice did not demonstrate hypercytokinemia like their constitutive counterparts (Fig. 2C compared to Fig. 2B). Furthermore, significant elevations in lung IAV viral load were not seen (Fig. 2D). Therefore, we concluded that IAV-induced hypercytokinemia in *Ndufs4* KO mice is primarily a consequence of increased viral load with systemic spread rather than immune cell–intrinsic factors. Using this same inhalation model, we previously showed that IAV can spread beyond the lungs, triggering systemic immune activation in mice (*17*).

### *Ndufs4* KO lung epithelium displays impaired bioenergetics and enhanced viral loads

**Fig. 3.**
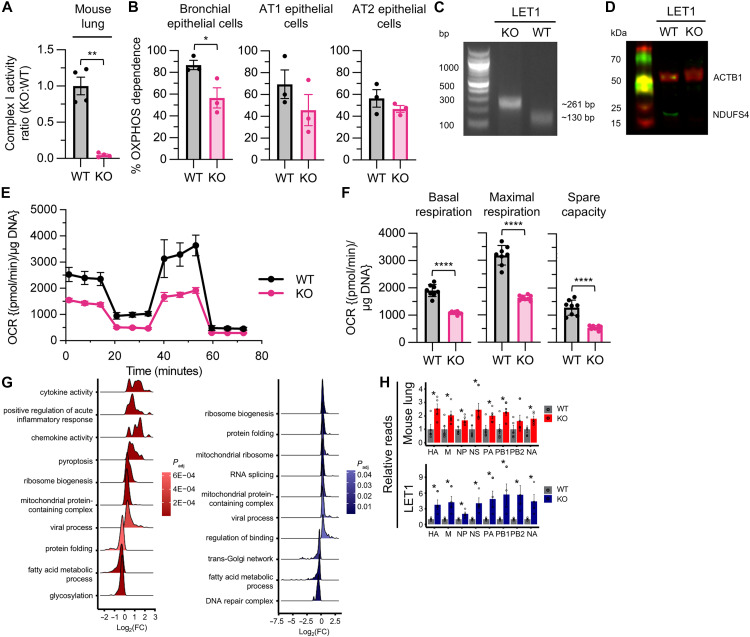
*Ndufs4* KO lung epithelium displays impaired bioenergetics and enhanced viral loads. (**A**) Complex I activity from WT and KO mice, *n* = 4 for WT and KO. (**B**) OXPHOS dependence (%) of bronchial, AT1, and AT2 epithelial cells isolated from UI WT and KO mouse lungs. Data were analyzed using unpaired *t* tests, *n* = 3 for WT and KO. (**C**) BccI RE digest of PCR products amplified from the CRISPR-edited region of genomic DNA revealed genotypes of WT and KO LET1 cells. (**D**) Near-infrared fluorescent Western blot of ACTB1 and NDUFS4 in WT and KO LET1 cells. (**E**) Mitochondrial stress test (MST) profile for WT and KO LET1 cells, *n* = 8 for WT and KO. (**F**) Mitochondrial function parameters, including basal respiration, maximal respiration, and spare capacity, calculated from MST profiles on WT and KO LET1 cells, *n* = 8 for WT and KO. Data in (A), (B), (E), and (F) are presented as means ± SD and were analyzed using unpaired *t* tests with Welch’s correction, unless otherwise indicated. (**G**) Ridge plots of significant GO pathways positively or negatively enriched through GSEA in IAV-infected KO versus WT mouse lung (left, red) or LET1 cells (right, blue). Representative GO pathways are shown. Color reflects the Benjamini-Hochberg adjusted *P* value, and *x* axis reflects the fold change (FC) of genes in the set. (**H**) Relative X31 viral reads for IAV gene segments HA, matrix (M), nucleoprotein (NP), nonstructural (NS), acidic polymerase (PA), basic polymerases 1 and 2 (PB1 and PB2, respectively), and NA between IAV-infected KO and WT mouse lung (red, top), and LET1 cells (blue, bottom).

Given that lung viral loads were not elevated in *CD4-* or *LysM-cre Ndufs4 KO* lungs, we reasoned that the increased viral burden observed in *Ndufs4* KO mice arises from factors intrinsic to the lung tissue. Because the lung epithelium is both a primary site of IAV infection and dependent on mitochondrial metabolism (*18*), we hypothesized that mitochondrial dysfunction in these cells may influence host-virus dynamics in a way that enhances viral infection. To explore this possibility, we first asked whether *Ndufs4* KO mice exhibit impaired mitochondrial function in the lung. Complex I activity in lung homogenates was depressed in *Ndufs4* KO relative to WT (Fig. 3A). To evaluate cellular bioenergetics, we quantified OXPHOS dependence in lung epithelial cell population subsets from *Ndufs4* KO lungs by flow cytometry using cell surface markers, a fluorescent ATP sensor, and oligomycin treatment. Flow cytometry showed reduced OXPHOS dependence in bronchial epithelial cells, with similar trends observed in alveolar type 1 (AT1) and type 2 (AT2) cell populations compared to WT (Fig. 3B).

After confirming that OXPHOS dysfunction extends to the lungs of *Ndufs4* KO mice, we developed an in vitro model using mouse lung epithelial type 1 (LET1) cells, which support IAV replication (*19*), to study host-pathogen interactions. Using CRISPR, we edited LET1 cells at exon 1 to generate a 4–base pair (bp) deletion, which resulted in a frameshift mutation and the introduction of a premature stop codon (fig. S2). PCR genotyping (Fig. 3C) and immunoblot analyses (Fig. 3D) confirmed the absence of *Ndufs4*. To verify OXPHOS deficiency, we next performed extracellular flux analysis. *Ndufs4* KO LET1 cells demonstrated reduced basal respiration, maximal respiration, and spare respiratory capacity compared to WT, indicative of impaired OXPHOS activity (Fig. 3, E and F), consistent with complex I deficiency.

To evaluate how closely our in vitro model mirrored the in vivo system, we performed RNA sequencing (RNA-seq) on *Ndufs4* KO lungs and CRISPR-edited *Ndufs4* KO LET1 cells. Initial principal components analysis (PCA), clustering, and differential expression analyses showed clear separation across genotypes and treatment conditions (i.e., IAV infection) in lung tissue and LET1 cells (fig. S3). Comparing either IAV-infected mouse lungs or LET1 *Ndufs4* KO against WT, we observed considerable transcriptional dysregulation in both models (fig. S4A). We next isolated genes that were either up-regulated or down-regulated in both lung and LET1 comparisons (fig. S4B), and pathway analysis of this gene set suggested significant increases in translation, death receptor, and IL-6 signaling pathways, whereas extracellular matrix organization was suppressed (fig. S4C and data S1). Upstream regulator analysis showed significant increased pathway activity of multiple antiviral mediators, including *Sting, Myd88*, *Tlr3*, *Rigi*, *Il1a*, and *Mavs* (fig. S4D and data S2). We next performed gene set enrichment analysis (GSEA) against gene ontology (GO) pathways for both mouse lung and LET1 cells (Fig. 3G). Both comparisons showed enrichment in translation and viral response, but as expected for in vivo conditions, lung tissue reflected a stronger enrichment of cytokine-related pathways. We next tested whether our in vitro model could support viral replication by analyzing viral reads in our RNA-seq data. *Ndufs4* KO lungs showed increased amplification of all viral gene segments compared to WT (Fig. 3H and data S3 and S4), a pattern that was also observed in LET1 *Ndufs4* KO cells. These findings confirm OXPHOS deficiency in *Ndufs4* KO lung tissue and establish a cellular model to study its effects in lung epithelial cells.

### Metabolic reprogramming to glycolysis in mitochondrial dysfunction

**Fig. 4.**
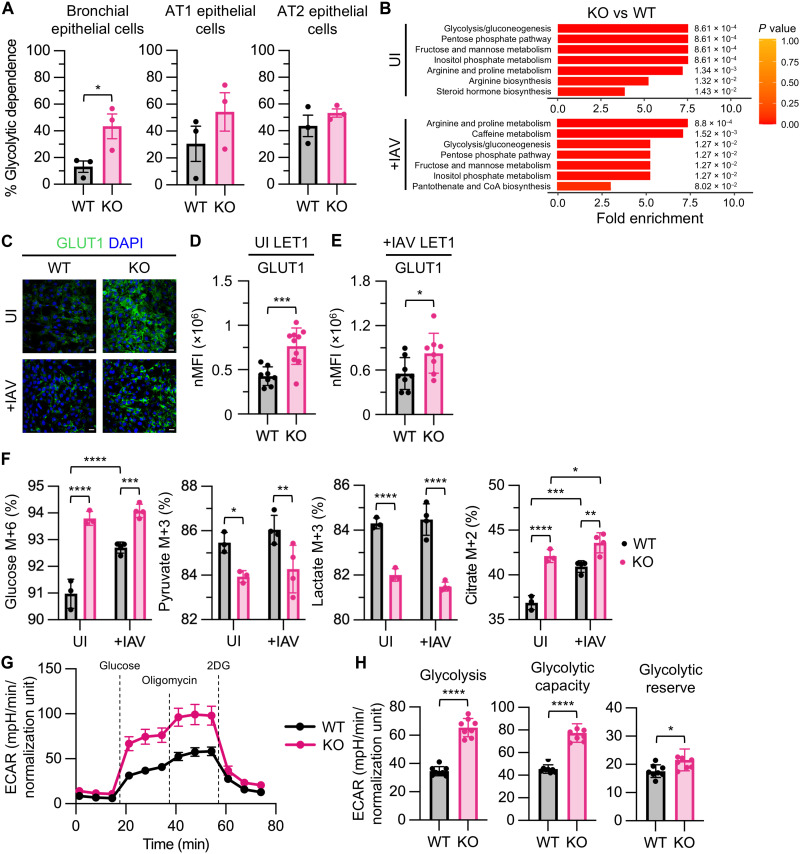
Metabolic reprogramming in *Ndufs4* KO enhances glycolysis. (**A**) Glycolytic dependence (%) of bronchial, AT1, and AT2 epithelial cells isolated from UI WT and KO mouse lungs. Data were analyzed using unpaired *t* tests, *n* = 3 for WT and KO. (**B**) Metabolic pathway enrichment analysis of untargeted metabolomics data from UI and IAV-infected KO versus WT LET1 cells, *n* = 6 for UI WT, UI KO, WT +IAV, and KO +IAV. (**C**) Confocal microscopy imaging and quantitation of GLUT1 nMFI in (**D**) UI and (**E**) IAV-infected WT and KO LET1 cells. Scale bars, 20 μm; *n* = 8 for UI WT and KO +IAV, *n* = 10 for UI KO, and *n* = 7 for WT +IAV. (**F**) Uniform ^13^C-glucose isotope labeling of metabolic intermediates in UI and IAV-infected WT and KO LET1 cells. Data were analyzed using an ordinary two-way ANOVA with uncorrected Fisher’s least significant difference (LSD) test, *n* = 3 for UI WT and UI KO, *n* = 4 for WT +IAV and KO +IAV. (**G**) Glycolysis stress test (GST) profile for WT and *Ndufs4* KO LET1 cells, *n* = 8 for WT and KO. (**H**) Glycolytic function parameters, including glycolysis, glycolytic capacity, and glycolytic reserve, calculated from GST profiles on WT and KO LET1 cells, *n* = 8 for WT and KO. Data in (A) and (D) to (H) are presented as means ± SD and were analyzed using unpaired *t* tests with Welch’s correction, unless otherwise indicated.

Although OXPHOS dysfunction was evident in *Ndufs4* KO lungs and LET1 cells, it remained unclear whether this bioenergetic impairment was sufficient to drive metabolic reprogramming toward glycolysis to maintain ATP production (*4*). Adaptive responses to OXPHOS deficiency have been well documented in the heart, brain, muscle, and liver in MtDs (*20*–*23*). To assess metabolic reprogramming, we measured glycolytic dependence in *Ndufs4* KO mouse lung epithelial cell population subsets by flow cytometry using cell surface markers, a fluorescent ATP sensor, and 2-deoxyglucose (2DG) treatment. Bronchial epithelial cells showed increased glycolytic dependence, with similar trends in AT1 and AT2 cell populations compared to WT (Fig. 4A). To address whether glycolysis was up-regulated in *Ndufs4* KO LET1 cells, we performed untargeted metabolomics on UI and IAV-infected *Ndufs4* KO LET1 cells. In UI *Ndufs4* KO LET1 cells, 193 metabolites were up-regulated, whereas 79 were down-regulated compared to WT (fig. S5A and data S5). In IAV-infected *Ndufs4* KO LET1 cells, 140 metabolites were up-regulated and 144 were down-regulated compared to WT (fig. S5B and data S6). Because metabolomics offers a snapshot of intermediary metabolism and cannot always be used to interpret the production or utilization of metabolites, we conducted metabolite set enrichment analysis (MSEA) to identify altered metabolic pathways for further study. In UI *Ndufs4* KO LET1 cells, the most enriched pathways included glycolysis/gluconeogenesis, the pentose phosphate pathway, fructose and mannose metabolism, and amino acid metabolism. Similar pathways were seen in IAV-infected *Ndufs4* KO LET1 cells (Fig. 4B).

Given the enrichment of glycolytic pathways in *Ndufs4* KO LET1 cells at baseline and during infection, we next assessed glucose uptake potential by measuring glucose transporter 1 (GLUT1) expression using immunofluorescence microscopy (Fig. 4C). Although GLUT1 levels remained consistent between UI and IAV-infected conditions in WT LET1 cells, *Ndufs4* KO LET1 cells exhibited higher levels of GLUT1 expression in both conditions (Fig. 4, D and E). To further characterize how glucose metabolism is altered in *Ndufs4* KO LET1 cells, we used stable isotope tracing with ^13^C-labeled glucose to track the fate of glucose-derived carbons. Glucose (M+6) uptake increased in WT cells during IAV infection as expected. Pyruvate (M+3) and lactate (M+3) labeling were similar under both conditions. Although glucose uptake was elevated in *Ndufs4* KO LET1 cells, downstream labeling of pyruvate (M+3) and lactate (M+3) was reduced (Fig. 4F). Notably, citrate (M+2) labeling increased in WT cells following infection, whereas *Ndufs4* KO cells showed elevations at baseline that rose even further with infection. Given the altered glucose metabolism observed in *Ndufs4* KO LET1 cells, we performed extracellular flux analysis to functionally assess glycolytic activity. *Ndufs4* KO LET1 cells showed elevated glycolysis, glycolytic capacity, and glycolytic reserve compared to WT (Fig. 4, G and H), confirming metabolic reprogramming to enhanced glycolysis.

### Loss of *Ndufs4* unleashes SIA biosynthesis through inadequate AMPK control

**Fig. 5.**
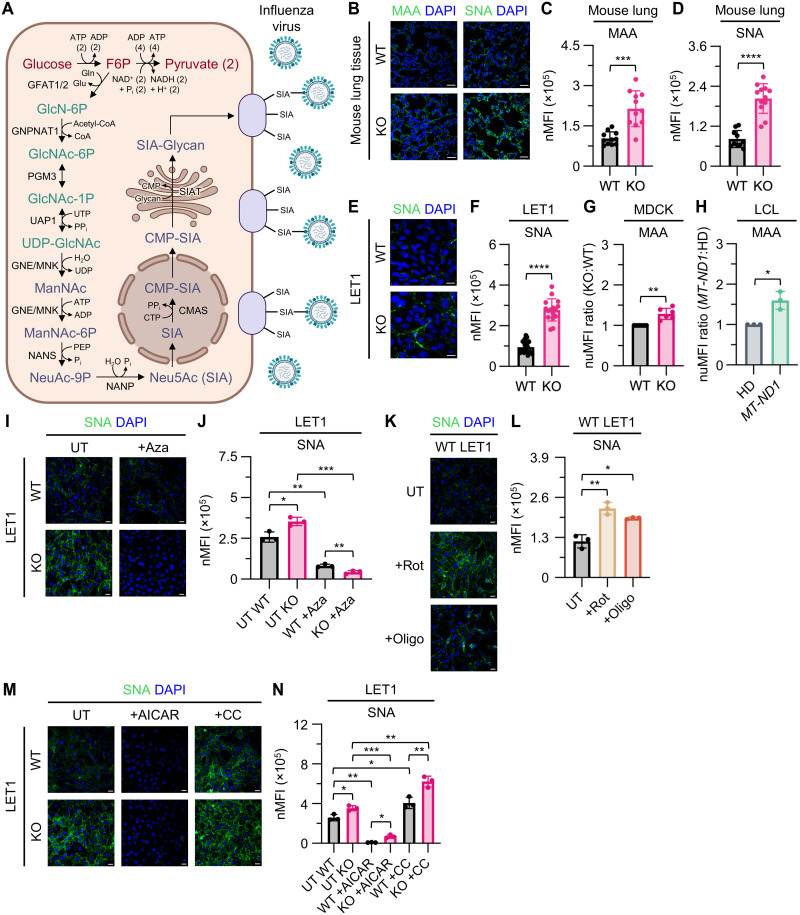
Complex I deficiency and mitochondrial dysfunction enhances SIA expression through impaired AMPK regulation. (**A**) Illustration of glycolytic link into the hexosamine pathway, SIA biosynthesis, and influenza attachment via SIA-glycans. (**B**) Confocal microscopy imaging and quantitation of (**C**) MAA and (**D**) SNA nMFI in UI WT and KO mouse lung tissue, *n* ≥ 10 for WT and *n* ≥ 11 for KO. (**E**) Confocal microscopy imaging and quantitation of (**F**) SNA nMFI in UI WT and KO LET1 cells, *n* = 17 for WT and *n* = 16 for KO. (**G**) Flow cytometry analysis of MAA lectin staining nuMFI ratios (KO:WT) in WT and KO MDCK cells, *n* = 6 for WT and KO. (**H**) Flow cytometry analysis of MAA lectin staining nuMFI ratios (*MT-ND1*:HD) in HD and *MT-ND1* pathogenic variant LCL cells, *n* = 3 for HD and *MT-ND1*. (**I**) Confocal microscopy imaging and quantitation of (**J**) SNA nMFI in UT and 3 μM azaserine-treated (+Aza) WT and KO LET1 cells, *n* = 3 for UT WT, UT KO, WT +Aza, and KO +Aza. (**K**) Confocal microscopy imaging and quantitation of (**L**) SNA nMFI in UT, 0.05 μM rotenone-treated (+Rot), and 0.1 μM oligomycin-treated (+Oligo) WT LET1 cells, *n* = 3 for UT, +Rot, and +Oligo. (**M**) Confocal microscopy imaging and quantitation of (**N**) SNA nMFI in UT, 0.25 mM AICAR-treated (+AICAR), and 1 μM CC-treated (+CC) WT and KO LET1 cells, *n* = 3 for UT WT, UT KO, WT +AICAR, KO +AICAR, WT +CC, and KO +CC. Scale bars in (B), (E), (I), (K), and (M), 20 μm. Data in (C), (D), (F) to (H), (J), (L), and (N) are presented as means ± SD and were analyzed using unpaired *t* tests with Welch’s correction, unless otherwise indicated.

Because glycolysis was elevated in *Ndufs4* KO lungs and LET1 cells, we next investigated whether metabolic pathways branching from glycolysis were also up-regulated. We focused on de novo SIA biosynthesis because sialylated glycoproteins serve as key receptors for IAV infection. Glycolysis supplies intermediates that feed into the hexosamine and SIA biosynthesis pathways, leading to the production of cell-surface glycoconjugates (*24*–*26*). The IAV hemagglutinin (HA) protein binds to SIA residues to mediate viral entry (Fig. 5A) (*27*). Given the increase in glycolysis, carbon diversion, and enrichment of fructose and mannose pathways (Fig. 4), we hypothesized that de novo SIA biosynthesis is enhanced in *Ndufs4* KO cells. To test this, we stained lung tissue from *Ndufs4* KO mice for α2,3-linked SIA (α2,3-SIA) and α2,6-linked SIA (α2,6-SIA) using *Maackia amurensis* (MAA) and *Sambucus nigra* (SNA) lectins, respectively (Fig. 5B). *Ndufs4* KO lungs showed increased staining for both α2,3-SIA and α2,6-SIA compared to WT (Fig. 5, C and D). We next validated these findings in vitro. *Ndufs4* KO LET1 cells also showed elevated α2,6-SIA levels compared to WT (Fig. 5, E and F). Similarly, *Ndufs4* KO Madin-Darby canine kidney (MDCK) cells, commonly used for cultivating IAV, also displayed higher α2,3-SIA levels relative to WT (Fig. 5G). To investigate whether increased SIA is a broader feature of MtDs, we analyzed a lymphoblastoid cell line (LCL) derived from a patient with a pathogenic *MT-ND1* variant (complex I deficiency). *MT-ND1* LCLs exhibited elevated α2,3-SIA levels by flow cytometry, similar to the increases observed in *Ndufs4* KO lung tissue and LET1 cells (Fig. 5H). To confirm that SIA biosynthesis in *Ndufs4* KO cells depends on de novo glycolytic input rather than salvage pathways, we inhibited glutamine:fructose-6-phosphate amidotransferase (GFAT), the rate-limiting enzyme of the hexosamine biosynthetic pathway, using azaserine. Azaserine treatment reduced surface SIA levels in *Ndufs4* KO LET1 cells, consistent with reliance on the de novo pathway (Fig. 5, I and J).

Having established that SIA biosynthesis depends on glycolytic input, we next investigated how cellular energy status regulates this pathway. To determine whether mitochondrial dysfunction alone was sufficient to enhance SIA production, we treated WT LET1 cells with the OXPHOS inhibitors rotenone and oligomycin. Both treatments substantially augmented surface SIA levels (Fig. 5, K and L). We then assessed whether adenosine monophosphate–activated protein kinase (AMPK), a central energy sensor, modulates SIA biosynthesis. AMPK normally restrains the hexosamine biosynthetic pathway and SIA production by inhibiting GFAT (*28*). Activation of AMPK with 5-aminoimidazole-4-carboxamide-1-β-D-ribofuranoside (AICAR) reduced SIA levels in both WT and *Ndufs4* KO LET1 cells (Fig. 5, M and N), indicating that the hexosamine and SIA biosynthesis pathways remain responsive to AMPK regulation despite OXPHOS dysfunction. Inhibition of AMPK with compound C (CC) increased SIA levels, with a more pronounced effect in *Ndufs4* KO cells (Fig. 5, M and N). This suggests that, although the hexosamine and SIA biosynthesis pathways remain sensitive to AMPK regulation, it is insufficient to tame the enhanced glycolytic flux through these accessory pathways in *Ndufs4* KO.

### Infection amplifies sialylation with enhanced IAV attachment in *Ndufs4* KO models

**Fig. 6.**
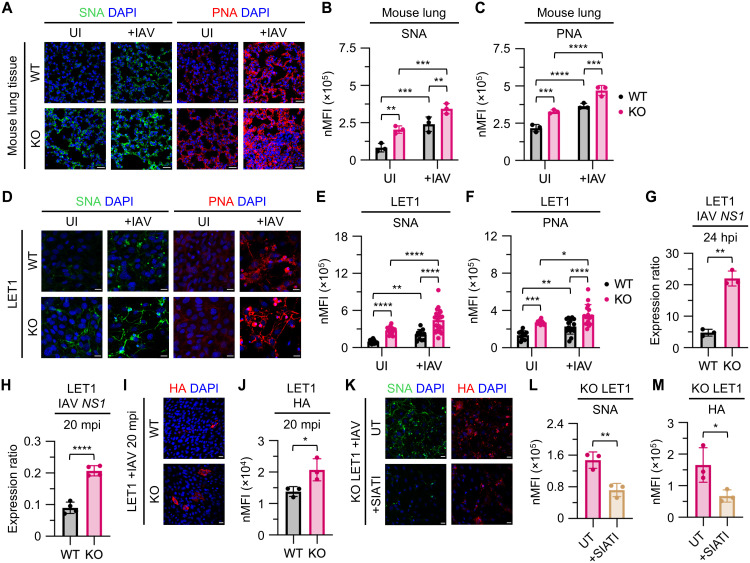
Enhanced SIA during IAV infection in *Ndufs4* KO mediates increased viral attachment. (**A**) Confocal imaging and quantitation of (**B**) SNA and (**C**) PNA nMFI in UI and IAV-infected WT and KO mouse lung tissue. Data were analyzed using ordinary two-way ANOVAs with uncorrected Fisher’s LSD multiple comparisons tests, *n* = 3 for UI WT, UI KO, +IAV WT, and +IAV KO. (**D**) Confocal microscopy imaging and quantitation of (**E**) SNA and (**F**) PNA nMFI in UI and IAV-infected WT and KO LET1 cells. Data were analyzed using ordinary two-way ANOVAs with uncorrected Fisher’s LSD multiple comparisons tests, *n* ≥ 10 for UI WT, *n* ≥ 11 for UI KO, *n* ≥ 12 for +IAV WT, and *n* ≥ 16 for +IAV KO. (**G**) Lung IAV *NS1* in WT and KO LET1 cells at 24 hpi, *n* = 3 for WT and KO. (**H**) IAV attachment *NS1* in WT and KO LET1 cells at 20 mpi, *n* = 4 for WT and KO. (**I**) Confocal imaging and quantitation of (**J**) HA nMFI in WT and KO LET1 cells at 20 mpi. Data were analyzed using an unpaired *t* test, *n* = 3 for WT and KO. (**K**) Confocal microscopy imaging and quantitation of (**L**) SNA and (**M**) HA nMFI in UT and SIATI-treated IAV-infected KO LET1 cells. Data were analyzed using unpaired *t* tests, *n* = 3 for UT and +SIATI. Scale bars in (A), (D), (I), and (K), 20 μm. Data in (B), (C), (E) to (H), (J), (L), and (M) are presented as means ± SD and were analyzed using unpaired *t* tests with Welch’s correction, unless otherwise indicated.

Building on our finding that OXPHOS dysfunction elevates surface SIA levels, we next investigated whether this increase enhances viral susceptibility during IAV infection. Prior studies in ferrets have shown that IAV infection induces marked up-regulation of both α2,3- and α2,6-SIA in bronchial epithelial cells (*29*), and emerging evidence indicates that IAV can suppress AMPK signaling to facilitate this biosynthetic shift (*30*, *31*), suggesting that infection itself may further amplify sialylation through metabolic dysregulation. To assess whether IAV amplifies SIA expression further in the context of OXPHOS dysfunction, we stained UI and IAV-infected lung tissues from *Ndufs4* KO and WT mice at 4 dpi using SNA to detect α2,6-SIA and peanut agglutinin (PNA), which binds exposed β-galactose residues characteristic of asialylated glycans (*32*). Compared to WT and UI *Ndufs4* KO lungs, IAV-infected *Ndufs4* KO tissues exhibited greater SNA staining, indicating IAV infection–induced up-regulation of α2,6-SIA (Fig. 6, A and B). PNA staining was also higher in IAV-infected *Ndufs4* KO lungs (Fig. 6, A and C), consistent with enhanced neuraminidase (NA) activity and viral particle release, which expose terminal β-galactose residues (*33*). Similar findings were observed in LET1 cells infected with IAV for 24 hours, which showed increased SNA and PNA staining (Fig. 6, D to F). This heightened susceptibility to IAV in *Ndufs4* KO LET1 cells was further supported by elevated viral loads at 24 hours postinfection (hpi), as measured by real-time RT-PCR targeting the IAV *NS1* gene segment (Fig. 6G).

Prompted by the viral infection-induced rise in surface SIA, we next investigated whether this change translates into enhanced IAV attachment in *Ndufs4* KO cells. LET1 cells were incubated with IAV for 20 min, corresponding to the early attachment phase prior to nuclear import and replication (*34*). Real-time RT-PCR analysis at this early time point revealed higher levels of IAV *NS1* in *Ndufs4* KO LET1 cells, indicating increased viral binding (Fig. 6H). Confocal microscopy and quantification of HA staining at 20 min postinfection (mpi) confirmed this result, showing greater viral attachment in *Ndufs4* KO cells compared to WT (Fig. 6, I and J). To confirm that this effect was mediated by elevated surface sialylation, we treated *Ndufs4* KO LET1 cells with a sialyltransferase inhibitor (SIATI), 3Fax-Peracetyl Neu5Ac. SIATI treatment reduced both SNA (Fig. 6, K and L) and HA (Fig. 6, K and M) staining, supporting a sialylation-dependent mechanism. Together, these findings demonstrate that elevated surface SIA in *Ndufs4* KO models, further amplified during IAV infection, promotes enhanced viral attachment.

### Metabolic targeting with dichloroacetate reduces viral load and inflammation in *Ndufs4* KO mice

**Fig. 7.**
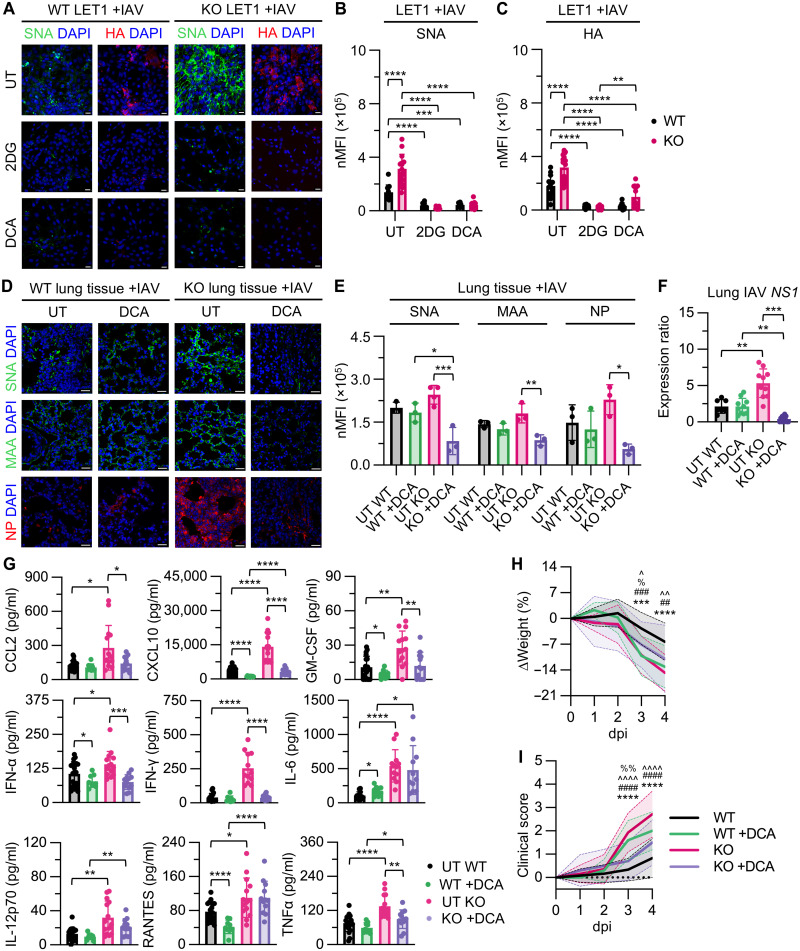
Targeting glycolysis reduces SIA and abrogates viral infection in *Ndufs4* KO mice. (**A**) Confocal microscopy imaging and quantitation of (**B**) SNA and (**C**) HA nMFI in UT, 2DG-treated, and DCA-treated IAV-infected WT and KO LET1 cells, *n* = 13 for UT WT, *n* ≥ 16 for UT KO, *n* = 16 for WT +2DG, *n* ≥ 18 for KO +2DG, *n* = 10 for WT +DCA, and *n* ≥ 13 for KO +DCA. (**D**) Confocal imaging and quantitation of (**E**) SNA, MAA, and NP nMFI in UT and DCA-treated IAV-infected WT and KO mouse lung tissue, *n* = 3 for UT WT, WT +DCA, KO +DCA, and *n* ≥ 3 for UT KO. (**F**) Lung IAV *NS1* in UT WT, DCA-treated WT, UT KO, and DCA-treated KO mice at 4 dpi. Data were analyzed using a Brown-Forsythe and Welch ANOVA test with Dunnett’s T3 multiple comparisons test, *n* = 7 for WT, *n* = 10 for WT +DCA and KO, and *n* = 13 for KO +DCA. (**G**) Plasma cytokine levels in UT WT, DCA-treated WT, UT KO, and DCA-treated KO mice at 4 dpi. Data were analyzed using unpaired *t* tests with Welch’s correction, *n* ≥ 18 for WT, *n* = 9 for WT +DCA, *n* ≥ 16 for KO, and *n* ≥ 11 for KO +DCA. DCA treatment of KO mice improves (**H**) body weight and (**I**) clinical scoring data during IAV infection, *n* = 12 for WT, *n* = 10 for WT +DCA and KO +DCA, and *n* ≥ 13 for KO. Scale bars in (A) and (D), 20 μm. Data in (B), (C), and (E) to (I) are presented as means ± SD and were analyzed using an ordinary two-way ANOVA with Tukey’s multiple comparisons test, unless otherwise indicated.

Having shown that OXPHOS dysfunction in *Ndufs4* KO models elevates glycolytic flux and surface sialylation, we next asked whether this metabolic shift could be therapeutically targeted. Because glycolysis fuels SIA biosynthesis and facilitates IAV binding, we tested whether metabolic modulation could reduce viral susceptibility in vitro and improve infection outcomes in vivo. We used 2DG, a glucose analog that blocks glycolysis upstream at phosphoglucose isomerase (*35*), and dichloroacetate (DCA), an inhibitor of pyruvate dehydrogenase kinase that promotes pyruvate oxidation via the tricarboxylic acid (TCA) cycle (*36*), thereby diverting glucose carbons away from glycolysis. Confocal imaging of IAV-infected WT and *Ndufs4* KO LET1 cells treated with 2DG or DCA revealed reduced SNA and IAV HA staining (Fig. 7, A to C), consistent with decreased surface sialylation and viral infection. On the basis of these in vitro results, we moved forward with DCA for in vivo studies, given its ability to selectively reroute glucose flux toward the TCA cycle without broadly inhibiting glycolysis or impairing ATP production. DCA has also been trialed in MtDs to reduce lactate accumulation by promoting pyruvate oxidation, thereby supporting its relevance in our model (*37*, *38*). P30 WT and *Ndufs4* KO mice received intraperitoneal DCA (50 mg/kg) injections at −1 and 2 dpi (fig. S6). In DCA-treated *Ndufs4* KO mice, confocal microscopy showed reduced SNA, MAA, and IAV NP staining in lung tissue relative to untreated (UT) controls (Fig. 7, D and E), whereas no significant changes were observed in DCA-treated WT mice. Consistently, real time RT-PCR analysis of lung tissue revealed reduced IAV *NS1* levels in DCA-treated *Ndufs4* KO mice but not in WT relative to UT controls (Fig. 7F). DCA also alleviated systemic hypercytokinemia in *Ndufs4* KO mice, reducing plasma levels of CCL2, CXCL10, GM-CSF, IFN-α, IFN-γ, and TNFα relative to UT *Ndufs4* KO mice (Fig. 7G). Although some cytokines were reduced in WT mice (e.g., CXCL10, GM-CSF, IFN-α, and RANTES), the effect was more limited. Notably, DCA treatment improved weight loss and clinical severity in *Ndufs4* KO mice (Fig. 7, H and I), whereas it worsened both parameters in WT mice at 3 and 4 dpi.

## DISCUSSION

MtDs have long been associated with increased susceptibility to infections, a risk typically attributed to immune dysfunction (*39*), poor airway clearance, or generalized physiological fragility. However, our findings suggest an additional, cell-intrinsic mechanism rooted in the respiratory epithelium. Using conditional KO models, we show that deletion of *Ndufs4* in T cells or macrophages does not replicate the heightened viral susceptibility seen in whole-body KO, pointing away from immune cell–driven pathology. Instead, we find that complex I deficiency in epithelial cells triggers metabolic allostasis, marked by increased glucose uptake and glycolytic flux. This reprogramming diverts carbons into the hexosamine and SIA biosynthesis pathways, resulting in elevated surface sialylation that enhances IAV binding and infection. Given that SIAs serve as entry receptors for a range of respiratory viruses, including coronaviruses, enteroviruses, and adenoviruses (*40*–*46*), this epithelial metabolic phenotype may represent a generalizable mechanism of susceptibility.

The regulation of this altered metabolic state centers on AMPK, a critical sensor of cellular energy status. Under normal conditions, AMPK activation suppresses anabolic pathways, including the hexosamine biosynthetic pathway, by phosphorylating and inhibiting GFAT, the first committed step toward UDP-GlcNAc and SIA production (*28*). In our model, pharmacologic AMPK activation suppressed SIA expression, whereas AMPK inhibition enhanced it, particularly in *Ndufs4* KO cells. Although energy depletion typically activates AMPK, chronic respiratory chain defects can blunt this activation, potentially through up-regulation of folliculin and other inhibitors (*47*). This impaired AMPK signaling diminishes the cell’s ability to restrain biosynthetic metabolism, enabling increased sialylation. AMPK also suppresses mammalian target of rapamycin complex 1 (mTORC1), a key driver of anabolic growth. In *Ndufs4* KO mice, mTORC1 hyperactivity has been reported, and rapamycin treatment improves survival and metabolic abnormalities (*48*, *49*). Although we did not directly assess mTORC1 in this study, the reciprocal regulation between AMPK and mTORC1 suggests that unopposed mTORC1 activity may further amplify SIA biosynthesis in MtDs. Broader pharmacologic modulation of AMPK or mTORC1 could represent a therapeutic avenue, although caution is warranted given their wide-ranging physiological roles.

In parallel to host-derived metabolic changes, IAV itself actively reprograms cellular metabolism to optimize replication. For example, the NS1 protein activates the PI3K/Akt pathway via direct interaction with the p85 subunit, enhancing glucose uptake and cell survival (*50*, *51*). IAV infection also up-regulates hypoxia-inducible factor 1α (HIF-1α), promoting glycolytic enzyme expression and accelerating glucose metabolism, thereby shifting energy production away from OXPHOS and toward glycolysis (*5*). This metabolic shift enhances flux through ancillary biosynthetic routes such as the pentose phosphate pathway, which supports nucleotide synthesis and redox balance through NADPH generation (*52*). IAV also impairs mitochondrial function directly via matrix (M) protein–mediated inhibition of OXPHOS (*53*), suppresses fatty acid β-oxidation, and increases lipid synthesis—all of which divert metabolic resources toward viral progeny production. Autophagy and host protein degradation further supply amino acids for viral protein synthesis (*52*). In the context of *Ndufs4* deficiency, where OXPHOS is already impaired and glycolysis is up-regulated at baseline, IAV-induced metabolic reprogramming exacerbates an already dysregulated state. The absence of AMPK restraint in *Ndufs4* KO cells removes a key brake on the hexosamine and SIA biosynthesis pathways, whereas viral signals through PI3K/Akt and HIF-1α intensify glucose-driven carbon flow into these routes. As a result, IAV infection triggers a disproportionately greater increase in surface sialylation in *Ndufs4* KO cells than in WT, driving enhanced viral attachment and replication.

Metabolic modulation presents a promising strategy for reducing infection risk in mitochondrial dysfunction. Although global inhibition of glycolysis is not viable due to its central role in cellular energetics, rerouting glycolytic carbon flow offers a more targeted intervention. DCA enhances pyruvate dehydrogenase complex activity by inhibiting pyruvate dehydrogenase kinase, thus promoting mitochondrial oxidation of glucose-derived pyruvate and reducing substrate availability for biosynthetic pathways such as SIA production. In *Ndufs4* KO models, both in vitro and in vivo, DCA treatment reduced surface sialylation, decreased viral burden, and attenuated systemic inflammation, all without compromising ATP production. DCA has been previously trialed in MtDs to reduce lactic acidosis and promote oxidative metabolism (*37*, *54*, *55*), underscoring its translational potential. However, DCA worsened infection outcomes in WT animals, likely due to disruption of metabolic flexibility. In cells with intact OXPHOS and glycolytic balance, enforced pyruvate oxidation may limit biosynthetic adaptability, revealing a narrow therapeutic window that depends on underlying metabolic context.

In summary, we identify mitochondrial dysfunction as a driver of increased viral susceptibility through deregulated glucose metabolism and enhanced SIA biosynthesis. Impaired AMPK activity and mTORC1 hyperactivation together establish a permissive metabolic state that favors viral attachment. Targeted metabolic intervention, such as glucose oxidation redirection with DCA, may provide a viable therapeutic strategy in settings of mitochondrial dysfunction. These findings offer a framework for understanding how intrinsic bioenergetic states shape susceptibility to viral infection and suggest potential treatment strategies for both rare MtDs and common chronic respiratory conditions.

## MATERIALS AND METHODS

### Animals

*Ndufs4*^+/−^ mice were originally obtained through the Jackson Laboratory (donated by R. Palmiter at the University of Washington, Seattle, WA, USA), B6.129S4-*Ndufs4^tm1.1Rpa^*/J mouse strain no. 027058 (*11*), and bred to produce *Ndufs4*^−/−^ KO offspring, which were weaned at 21 days of age and housed thereafter with *Ndufs4*^+/−^ and/or *Ndufs4*^+/+^ WT littermates for warmth (*56*, *57*). Additional strains originally obtained through the Jackson Laboratory, include *CD4-* and *LysM-cre* mice, B6.Cg-Tg(Cd4-cre)1Cwi/BfluJ (strain no. 022071) and B6.129P2-*Lyz2^tm1(cre)Ifo^*/J (strain no. 004781), respectively, which were bred with B6.129S4-*Ndufs4^tm1Rpa^*/J (strain no. 026963) mice to produce *CD4-* and *LysM-cre* conditional *Ndufs4* KO mice. Genotyping of *Ndufs4* alleles, *CD4-cre*, and *LysM-cre* was conducted using recommended methods from the Jackson Laboratory. Mice were housed in a pathogen-free facility and had access to Purina Lab Diet rodent chow, product no. 5LL2, and autoclaved reverse osmosis water. Mice were kept in a temperature (22° ± 2°C) and humidity (30 to 70%) controlled environment with a 12-hour/12-hour light/dark cycle. Cages housing *Ndufs4*^−/−^ KO mice were provided food on the floor every day following weaning. *Ndufs4*^−/−^ KO mice were euthanized if they lost 20% of their maximum body weight, reported to be immobile, or found to be moribund (*56*, *57*). All animal care and procedures were authorized and approved by the Animal Care and Use Committee of the National Human Genome Research Institute under Animal Study Proposal G-11-3. All experiments were performed according to relevant guidelines and regulations.

### IAV infection and CSS

Mouse adapted human influenza virus A/X/31 (X31) subtype H3N2 was used for infections. Male and female mice (*Ndufs4*^−/−^ KO and *Ndufs4*^+/+^ WT) were exposed to aerosolized (Glas-Col) 8.55 × 10^10^ focus-forming units (FFU) in 7 ml of water. After infection, mice were monitored for weight loss and infection every 24 hours. A CSS system (table S1) was used to assess illness in IAV-infected mice (*58*). Expression of IAV *NS1* in the lungs of infected mice was determined by real-time RT-PCR. To investigate the effect of DCA on in vivo IAV infection, mice were administered DCA (50 mg/kg) by intraperitoneal injection at −1 and 2 dpi (fig. S6). LET1 cells were infected with 1% (v/v) X31 (8.55 × 10^9^ FFU/ml) in high glucose Dulbecco’s modified Eagle’s medium (DMEM) (catalog no. 12430054, Thermo Fisher Scientific) without fetal bovine serum (FBS) for 2 hours in the absence of exogenous trypsin at 37°C. After incubation with virus, media were aspirated, and cell monolayers were washed once with 1X phosphate-buffered saline (PBS). Followed by refreshment with complete cell culture media [high glucose DMEM containing 10% (v/v) FBS and penicillin-streptomycin (100 U/ml; catalog no. 15140122, Thermo Fisher Scientific)] and incubation for an additional 22 hours at 37°C.

To investigate the effects azaserine (catalog no. A1164-.5MG, MilliporeSigma), AICAR (catalog no. A9978-5MG, MilliporeSigma), and CC (catalog no. 171260-1MG, MilliporeSigma) on GFAT and AMPK modulation of SIA biosynthesis, WT and *Ndufs4* KO LET1 cells were seeded at 2.1 × 10^5^ cells per well in 12-well plates in 1 ml of complete LET1 cell culture media containing 3 μM azaserine, 0.25 mM AICAR, or 1 μM CC and cultured for 24 hours.

A subset of LET1 IAV infection experiments was conducted with an infection period of 20 min, after which cell monolayers were washed and collected for either RNA extraction or confocal microscopy.

To examine the effect of 3Fax-Peracetyl Neu5Ac (SIATI, catalog no. 566224-10MG, MilliporeSigma) on in vitro IAV infection, *Ndufs4* KO LET1 cells were seeded at 2.5 × 10^4^ cells per well in 12-well plates in 1 ml of complete LET1 cell culture media containing SIATI (11 μg/ml) and cultured for 72 hours. Subsequently, SIATI-treated LET1 cells were infected with X31 as described above in the presence of SIATI (11 μg/ml). Following incubation with virus, media were aspirated, cells were washed, and media were refreshed as described above; however, SIATI (11 μg/ml) was added to refreshment media.

In addition, to investigate the effect of 2DG and DCA on in vitro IAV infection, LET1 cells were infected with X31 as described above in the presence of 1.5 mM 2DG or 50 mM DCA. Following incubation with virus, media were aspirated, cells were washed, and media were refreshed as described above; however, 1.5 mM 2DG or 50 mM DCA was added to refreshment media.

### Quantification of *NS1* expression

Lung tissue samples were homogenized using a TissueRuptor (Qiagen) handheld rotor-stator homogenizer in PBS. Total RNA was extracted from clarified lung tissue homogenates using an RNeasy Mini Kit (Qiagen). Reverse transcription of 500 ng of RNA to cDNA was performed using an iScript cDNA synthesis kit (Bio-Rad Laboratories). Real-time RT-PCR was carried out in 20 μl using SYBR Green Supermix (Bio-Rad Laboratories) or TaqMan gene-expression assays [murine β-actin (catalog no. Mm01205647_s1), Thermo Fisher Scientific]. Reactions were thermocycled and quantitated with a CFX96 Real-Time PCR System (Bio-Rad Laboratories). All reported lung IAV load *NS1* expression ratios (2^−∆Cq^) are relative to *Actb*.

### IAV lung titering

An FFU test was used to determine viral titer as described by Payne *et al.* (*59*), which is a modified form of median tissue culture infectious dose (TCID_50_). This assay was performed with little modification from the original assay. In brief, MDCK cells were seeded at a density of 1 × 10^4^ cells per well in a 96-well plate. To perform the FFU titer test, 10-fold serial dilutions of clarified lung tissue homogenates were prepared and used to infect MDCK cell monolayers at 37°C. After 2 hours, the inoculum was aspirated, and the MDCK cells were rinsed with PBS followed by addition of fresh complete cell culture media with incubation at 37°C, 5% CO_2_ for 72 hours. After incubation, MDCK cells were rinsed with PBS and fixed for 15 min in 4% paraformaldehyde (PFA) followed by blocking for 1 hour in 1% bovine serum albumin (BSA) and overnight incubation with IAV-specific NP monoclonal antibody (Ab) (catalog no. MA1-7322, Thermo Fisher Scientific) at 4°C. The primary Ab was aspirated the following day followed by three PBS washes. A Zeiss Axiovert A1 fluorescent microscope was used to visualize and count infection foci using a 10X objective lens. Titers were calculated as FFU/ml culture supernatant using the following formula: FFU = {[(*F*_1_ × *D*_1_) + (*F*_2_ × *D*_2_)/2]/vol} × 1000, where *F*_1_ is the total number of infectious virus foci in the maximum dilution, *D*_1_ is the dilution factor of *F*_1_, *F*_2_ is the total number of infectious virus foci one dilution higher than *F*_1_, *D*_2_ is the dilution factor of *F*_2_, and vol is the total volume taken from clarified lung tissue homogenates to make the serial dilutions.

### Lung histology and pathological scoring

Lung tissue was perfused with PBS prior to immersion fixation in 10% formalin for 24 hours. Following fixation, lung tissues were rinsed with deionized water and stored in 70% (v/v) ethanol at 4°C prior to being submitted to HistoServ Inc. (Germantown, MD) for paraffin embedding, sectioning, and hematoxylin and eosin (H&E) staining. Slide imaging and digitization was conducted using an Axio Scan Z.1 (Zeiss) slide scanner. The H&E-stained slides were coded and evaluated by an independent pathologist blinded to experimental groups. Each tissue section was scored based on a scale of 0 to 7 with increments of 1, with 0 as no inflammation or notable lesions and 7 as the highest degree of inflammation, immune cell tissue infiltration, and/or presence of severe lesions.

### LDH assay

An LDH assay kit (catalog no. MAK066, MilliporeSigma) was used to measure LDH activity levels in BAL fluid samples, which were collected via intratracheal administration of 0.5 l of ice-cold PBS followed by centrifugation at 800*g* for 10 min and storage at −80°C until analysis, following the manufacturer’s instructions.

### Quantification of plasma and lung homogenate cytokines

Cytokine concentrations were determined based on the manufacturer’s protocols for the LEGENDplex Mouse Anti-Virus Response Panel (BioLegend). In brief, peripheral blood from anesthetized mice was collected using heparinized capillary tubes (catalog no. 2501, Kimble Chase) prior to centrifugation at 1800*g* for 10 min to separate plasma. Plasma was then pipetted off and stored at −80°C until use. Clarified lung tissue homogenate samples were prepared using a TissueRuptor (Qiagen) handheld rotor-stator homogenizer in PBS followed by centrifugation at 800*g* for 10 min and storage at −80°C until analysis.

Frozen plasma and lung homogenate samples were completely thawed and all assays were performed in 96-well V-bottom plates. Once thawed, 12.5 μl of plasma samples were added to wells along with 12.5 μl of assay buffer and 12.5 μl of beads. Plates were then sealed with film, covered with aluminum foil and placed on an orbital shaker set to 800 rpm for 1 hour at room temperature. Plates were then centrifuged at 250*g* for 5 min, the supernatant was discarded by quick and forceful inversion, and pelleted beads were washed twice with 0.2 ml of 1X wash buffer. Following washes, 12.5 μl of detection Ab was added to each well, plates were resealed, covered with foil, and shaken at 800 rpm for 30 min at room temperature. Then, 12.5 μl of streptavidin-PE (SA-PE) was added to each well, and plates were resealed, covered with foil, and shaken at 800 rpm for 15 min at room temperature. After incubation, plates were centrifuged at 250*g* for 5 min, washed once with 0.2 ml of 1X wash buffer, and pelleted beads were resuspended in 0.18 ml of 1X wash buffer. Samples were analyzed using a CytoFLEX LX flow cytometer (Beckman Coulter), and data were analyzed using the manufacturer-supplied LEGENDplex Data Analysis Software Suite (BioLegend).

### Generation of CRISPR-edited *Ndufs4* KO mouse LET1 cells

CRISPR editing of WT LET1 cells to generate *Ndufs4* KO LET1 cells was conducted by the NHLBI iPS and Genome Engineering Core. In brief, 7 × 10^5^ million LET1 cells were transfected with premixed 20 μg of HiFiCas9 V3 (IDT #1081061) and 175 pmol of sgRNA1 CCUGGAUGGAACUCUACAGA or sgRNA2 CCGUCUGUAGAGUUCCAUCC (Synthego) using Nucleofector 4D with buffer SF and program DS-150 (Lonza) and then plated each to a 6-well plate with 3 ml of DMEM + 10% FBS. Transfected cells were cultured in a 32°C incubator with a medium change 24 hours after transfection. Three days after transfection, single-cell subcloning was done using a BD FACSMelody cell sorter to sort 1 cell per well to a 96-well plate with DMEM + 10% FBS. Single-cell clones were expanded at 37°C with one half medium change every other day for 2 to 3 weeks. Targeted sequence was amplified by genomic PCR using Phusion HS II polymerase supplemented with 1 M betaine (forward primer: TCTGAAGGCTTGCAAACGGA; reverse primer: CGCTGTAACCTTCCGATGGA) and sequenced using primer GGCTTGCAAACGGAATCTGG. The Sanger sequencing results were analyzed by ICE software (Synthego). Clones with frameshift mutations on all alleles were selected as complete KO clones; clones with both WT and frameshift mutation alleles were selected as heterozygous KO clones; clones with WT allele only were selected as isogenic control WT clones.

### Cell culture

MDCK and LET1 cells were cultured in complete cell culture media, maintained at 37°C, 5% CO_2_, passaged using brief incubation with 0.05% (v/v) trypsin-EDTA and subcultured every 2 to 3 days. Healthy donor (HD) and MtD patient-derived LCL cell lines were cultured in RPMI supplemented with 10% (v/v) FBS, 1 mM sodium pyruvate, 1X nonessential amino acids, 1X glutamine, and 1X penicillin-streptomycin and maintained at 37°C, 5% CO_2_. A subset of experiments was conducted on WT LET1 cells treated with a complex I inhibitor, rotenone, or a complex V inhibitor, oligomycin. In brief, WT LET1 cells were seeded at 3 × 10^5^ cells per well in 12-well plates and were cultured for 24 hours in 1 ml of complete cell culture media containing either 0.05 μM rotenone or 0.1 μM oligomycin followed by washing and fixation for confocal microscopy.

### Complex I activity assay

Mitochondrial complex I activity was measured using an enzyme assay kit following the manufacturer’s protocol (catalog no. ab109721, Abcam). Briefly, 100 μg of lung tissue was homogenized in cold PBS using a Dounce homogenizer. Protein concentration was determined using a BCA protein assay kit (catalog no. 23225, Thermo Fisher Scientific). Equal amounts of protein from each sample were extracted using the detergent provided in the kit and then loaded onto the assay plate. After a 3-hour incubation at room temperature, the plate was washed three times with buffer. Subsequently, 200 μl of assay solution was added to each well, and optical density at 450 nm (OD_450_) was measured in kinetic mode for up to 30 min.

### Genotyping CRISPR-edited *Ndufs4* KO mouse LET1 cells

Genomic DNA isolates were prepared using a GeneJET genomic DNA purification kit (catalog no. K0721, Thermo Fisher Scientific) and manufacturer-provided protocols. PCR primers (LET1 forward primer: CCCGAGGAAGGGTCAAGGGA; LET1 reverse primer: CGTACTCGCATCCTGGCGTT) were designed to amplify a region of DNA spanning ~130 bp upstream and downstream of the 4-bp deletion site in the *Ndufs4* KO LET1 cells to generate a PCR product of ~260 bp. For PCR, 10 μl of MyTaq mix (catalog no. BIO-25042, Meridian Bioscience), 0.8 μl of 10 μM LET1 forward primer, 0.8 μl of 10 μM LET1 reverse primer, 4 μl of genomic DNA isolate, and 4.4 μl of nuclease free water were mixed in thin-walled PCR tubes followed by touch-down PCR cycling protocol consisting of (stage 1) 2 min at 94°C, (stage 2) 10 cycles of 45 s at 94°C, 1.5 min at 65°C (decreasing by 0.5°C each cycle), and 68°C for 1 min, (stage 3) 28 cycles of 45 s at 94°C, 1.5 min at 60°C, and 45 s at 72°C, (stage 4) 5 min at 72°C and indefinite hold at 10°C on a SimpliAmp Thermal Cycler (Applied Biosystems by Thermo Fisher Scientific) prior to storage at −20°C. Restriction enzyme (RE) digest of PCR products was conducted using BccI (catalog no. R0704S, New England Biolabs) and manufacturer-provided instructions. RE-digested PCR products were then analyzed directly using 2% (w/v) agarose gel electrophoresis in 1X tris-acetate-EDTA (TAE) buffer (catalog no. 1610743, Bio-Rad) and 3 × 10^−5^% (v/v) ethidium bromide (catalog no. 46067-50ML-F, Sigma-Aldrich) followed by imaging using a Gel Doc EZ Imager (Bio-Rad).

### Near-infrared fluorescent Western blotting

LET1 cells were lysed using mammalian protein extraction reagent (M-PER, Thermo Fisher Scientific) supplemented with protease and phosphatase inhibitors. Total protein was quantified using a Bradford assay and separated by electrophoresis on Mini-PROTEAN TGX 10% polyacrylamide gels (Bio-Rad). Proteins were transferred to nitrocellulose membranes using a Trans-Blot Turbo Transfer System (Bio-Rad) according to the manufacturer’s instructions. Membranes were blocked in Intercept Blocking Buffer (LI-COR Biosciences) for 1 hour at room temperature. They were then incubated with β-Actin Mouse monoclonal Ab (catalog no. AC004) and *NDUFS4* Rabbit monoclonal Ab (catalog no. A8691) diluted in 5% (w/v) BSA overnight at 4°C. After washing with 0.05% (v/v) Tween 20, membranes were incubated with IRDye-conjugated secondary antibodies for 1 hour at room temperature. Protein signals were detected using an Odyssey CLx infrared imaging system (LI-COR Biosciences).

### Mitochondrial stress test and glycolysis stress test assays

Oxygen consumption rate (OCR) and extracellular acidification rate (ECAR) were measured using a Seahorse XF96 analyzer (Agilent). WT and *Ndufs4* KO LET1 cells were seeded at 1 × 10^4^ to 2 × 10^4^ cells per well in a Seahorse XF96 well plate and cultured overnight in complete cell culture media at 37°C, 5% CO_2_. OCR and ECAR were determined using the Seahorse XF Mito Stress (catalog no. 103015-10) and Glycolysis Stress (catalog no. 103020-100) Test kits, respectively, according to the manufacturer’s standard protocols. OCR and ECAR values were recorded and calculated by the Seahorse XF96 Wave software (v2.6.3), which were then further normalized to DNA content. XF96 well plate DNA content was determined using a CyQUANT cell proliferation assay kit (catalog no. C7026, Thermo Fisher Scientific) and manufacturer-recommended protocol.

### RNA-seq analysis

Lung tissue samples were homogenized using a TissueRuptor (Qiagen) handheld rotor-stator homogenizer in PBS. Total RNA was extracted from clarified lung tissue and LET1 cell homogenates using an RNeasy Mini Kit (Qiagen). RNA-seq was performed by an outside commercial laboratory (Novogene, Sacramento, CA). mRNA was purified using poly-T oligo-attached magnetic beads. First strand cDNA was synthesized with random hexamer primers, followed by second strand cDNA synthesis using dTTP (3′-deoxythymidine 5′-triphosphate). Libraries then underwent end repair, A-tailing, adapter ligation, size selection, amplification, and purification. Sequencing was performed on the Illumina NovaSeq 6000 with 150-bp paired-end reads. Reads were aligned to reference genome mm10 with Hisat2 v2.0.5, and raw read counts were determined using FeatureCounts v1.5.0-p3. Raw count normalization and differential expression analysis was performed using DESeq2 1.42.0 (*60*). Volcano plots were prepared using EnhancedVolcano 1.20 with apeglm fold change shrinkage (*61*). For subsequent analysis, genes were considered significant if *P*_adj_ < 0.05 and log_2_ fold change > |0.263| where applicable. For viral read analysis, X31 sequences were downloaded through NCBI Virus using taxid:132504, 2006 sequences. fastq files were then aligned to the mouse genome using salmon (*62*), and unmapped sequences were collected and mapped to the X31 viral genome with Magic-BLAST (*63*). Reads mapping to each viral gene were then counted with salmon.

### Gene set enrichment analysis

Gene sets were ranked by t-stat determined by DESeq2 prior to enrichment analysis. GSEA and visualization were performed with clusterProfiler 4.11.0 (*64*).

### Lung tissue fixation, paraffin embedment, sectioning, and antigen retrieval for immunofluorescence

Lung tissue was perfused with PBS prior to immersion fixation in 10% formalin for 24 hours. Following fixation, lung tissues were rinsed with deionized water and stored in 70% (v/v) ethanol at 4°C prior to being submitted to HistoServ Inc. (Germantown, MD) for paraffin embedding and sectioning. Paraffin-embedded lung tissue sections were deparaffinized and rehydrated by immersing them in three changes of xylene for 3 min each, followed by a graded ethanol series (100% for 4 min, 95% for 1 min, and 70% for 1 min). The sections were then rinsed in cold water and placed in Coplin jars containing 1X antigen retrieval buffer (catalog no. 00-4955-58, Thermo Fisher Scientific). The jars were subsequently heated in an IHC Tek steamer (catalog no. NC1314441, Fisher Scientific) for 40 min. Afterward, the slides were allowed to cool completely in buffer overnight. The following day, immunostaining was conducted (*65*).

### Immunofluorescence staining and confocal microscopy

LET1 cells were cultured and infected as described above on sterile glass coverslips in 12-well plates at 60 to 80% confluency depending on the experimental necessity and time point. After the required time point, UI and IAV-infected cells were washed twice with tris-buffered saline (TBS) and fixed for 15 min in 4% PFA in TBS. Fixed LET1 cells and antigen-retrieved lung tissue sections were blocked and permeabilized for 1 hour in TBS with 0.1% (v/v) Triton X-100, 3% (w/v) BSA, and 5% (v/v) FBS. After blocking, cells were incubated overnight at 4°C with primary Ab or with lectin dyes using manufacturer-recommended dilutions. After three TBS washes, cells were incubated for 1 hour at room temperature with respective Alexa Fluor–conjugated secondary Abs (1:1000 diluted in TBS) (Thermo Fisher Scientific). Fixed cells and lung tissue sections were then counterstained with Prolong Gold Antifade DAPI (4′,6-diamidino-2-phenylindole) after three washes of TBS (Thermo Fisher Scientific). Microscopic images were captured using a 25X objective on a Zeiss LSM 880 confocal microscope. ImageJ software was used for image analysis and quantitation (*66*). All reported confocal microscopy quantitation immunofluorescence MFI data are normalized to cell number (nMFI).

### Antibodies and dyes

The antibodies and dyes used are as follows: GLUT1 (catalog no. ab115730, Abcam), LIVE/DEAD fixable aqua dead cell stain kit (catalog no. L34966, Thermo Fisher Scientific), CD24 (M1/69), eFluor 450 (Thermo Fisher Scientific), Podoplanin (eBio8.1.1), PerCP-eFluor 710 (Thermo Fisher Scientific), CD326 (EpCAM, clone G8.8), Alexa Fluor 700 (Thermo Fisher Scientific), CD31 (PECAM-1, clone 390), NovaFluor Red 755 (Thermo Fisher Scientific), *M. amurensis* lectin (MAA/MAL II) (catalog no. 21511103-1, Bioworld), *S. nigra* lectin (SNA, EBL) (catalog no. Fl-1301-2, Vector laboratory), PNA (catalog no. Cl-1075-1, Vector laboratory), anti–influenza A virus HA (a kind gift from the Yewdell lab, NIH), anti–influenza A virus NP (catalog no. BE0159, Biocell), Alexa Fluor 488 Goat anti-mouse (catalog no. A11029, Thermo Fisher Scientific), Alexa Fluor 647 Goat anti-mouse (catalog no. A21235, Thermo Fisher Scientific), and ProLong Gold Antifade Mountant with DNA Stain DAPI (catalog no. P36931, Thermo Fisher Scientific).

### Untargeted metabolomics

After 22-hour incubation following X31 infection as described above, cell culture media were aspirated, and LET1 cell monolayers were washed twice with prechilled (4°C) 1X PBS, gently collected using a cell scraper, and centrifuged at 400*g* for 5 min at 4°C. The supernatant was discarded, and cells were washed twice more using prechilled 1X PBS and subsequent centrifugation at 1000*g* for 5 min at 4°C. Cells were counted, transferred to 2-ml centrifuge tubes, and centrifuged at 1000*g* for 10 min at 4°C, the supernatant was discarded, and samples were flash frozen in liquid N_2_ followed by storage at −80°C. Frozen LET1 cell pellets were sent for untargeted metabolomics analyses by Novogene Corporation Inc. (Woburn, MA).

### Mitochondrial/energy flow cytometry metabolic dependence assay

Pulmonary epithelial cells, including bronchial epithelial cells (CD45.2^−^ CD326^+^ CD24^+^), AT1 epithelial cells (CD45.2^−^ CD326^+^ CD24^−^ PDP^lo^), and AT2 epithelial cells (CD45.2^−^ CD326^+^ CD24^−^ PDP^hi^) (*67*), were isolated from mouse lungs following a modified version of the protocol described in (*68*), originally developed for single-cell transcriptomics. In brief, mice were euthanized by CO_2_ inhalation. Lungs were carefully excised, rinsed in cold PBS, and transferred into a digestion solution containing collagenase CLS II (750 IU/ml, Sigma-Aldrich), DNase I (1 mg/ml, Sigma-Aldrich), and dispase (5,000 U/ml, Corning) prepared in RPMI with actinomycin D (2 mg/ml) to minimize ex vivo stress-induced gene expression. Tissue was mechanically dissociated by mincing with forceps and incubated for 30 min at 37°C under gentle agitation. Digestion was stopped by addition of cold 1% (w/v) BSA in PBS, and the resulting cell suspension was filtered through a 70-μm cell strainer, followed by red blood cell lysis using 1× RBC lysis buffer (Santa Cruz Biotechnology). After centrifugation and washing, cells were resuspended in 0.04% (w/v) BSA in PBS and filtered through a 40-μm cell strainer.

For flow cytometry analysis, cells were stained for 30 min at 37°C using the following antibodies: CD24 (M1/69), eFluor 450 (Thermo Fisher Scientific); Podoplanin (eBio8.1.1), PerCP-eFluor 710 (Thermo Fisher Scientific); CD326 (EpCAM, clone G8.8), Alexa Fluor 700 (Thermo Fisher Scientific); and CD31 (PECAM-1, clone 390), NovaFluor Red 755 (Thermo Fisher Scientific). Dead cells were excluded using the LIVE/DEAD fixable aqua dead cell stain kit (Thermo Fisher Scientific) according to the manufacturer’s instructions. Conditions tested to determine metabolic dependence were control (CO), oligomycin (10 μM, O), 2DG (100 mM, DG), etomoxir (100 μM, E), and a combination of all treatments (O + DG + E). The corresponding drug concentrations were added, followed by incubation for 30 min at 37°C, 5% CO_2_. After this, the fluorescent ATP sensor (1 μM ATP-Red, Sigma-Aldrich) was added to the cell suspension and incubated for 30 min at 37°C, 5% CO_2_ along with Ab staining. Samples were acquired on a CytoFLEX LX flow cytometer (Beckman Coulter), and data were analyzed using FlowJo v10 (*69*). The following calculations were then performed based on the results

$$\begin{array}{c} \%\text{Glycolytic dependence}=\\ \left(\text{CO}\;\text{MFI}-O\;\text{MFI}\right)/\left(\text{CO}\;\text{MFI}-\text{All}\;\text{MFI}\right)\times 100 \end{array}$$

$$\begin{array}{c} \%\text{OXPHOS dependence}=\\ \left(\text{CO}\;\text{MFI}-\text{DG}\;\text{MFI}\right)/\left(\text{CO}\;\text{MFI}-\text{All}\;\text{MFI}\right)\times 100 \end{array}$$

### Stable isotope metabolomics

Following X31 infection of LET1 cells as described above, media were replaced with media containing uniformly ^13^C-labeled analog ([U-^13^C]glucose; Cambridge Isotope Laboratories). Cells were cultured for an additional 22 hours. Then, plates were placed on ice, media were aspirated, cells were quickly rinsed with ice-cold normal saline solution and then overlaid with 0.5 ml of 80% (v/v) methanol. Cells were scraped, transferred to Eppendorf tubes, and stored at −80°C prior to being subjected to three rounds of alternating freeze-thaw cycles between liquid N_2_ and a 37°C water bath. Afterward, lysates were centrifuged at >10,000 rpm for 10 min at 4°C, and supernatants were transferred to new tubes and stored at −80°C until ready to ship. Frozen extracts were sent for stable isotope analyses by UT Southwestern Medical Center (Dallas, TX).

### Flow cytometry protocol for MDCK and LCL cells

Single-cell suspensions of cell lines were prepared by collection using cold gentle cell dissociation reagent (1X dPBS containing 2 mM EDTA) followed by staining with LIVE/DEAD aqua dead cell stain (1:1000 dilution) and MAA lectin (1:40 dilution) in 1X dPBS containing 1% (v/v) FBS and 0.05% (w/v) sodium azide (FACSB). Stain incubations were conducted for 30 to 60 min at 4°C followed by washing with FACSB. Data were acquired on a CytoFLEX LX flow cytometer and analyzed using CytExpert software (Beckman Coulter). All reported flow cytometry MAA lectin staining MFI data are normalized to unstained (nuMFI) controls.

### Statistical analysis

Statistical significance for all datasets was determined using GraphPad Prism v10 or R 4.3.2 with the following statistical indications: *P < 0.05, **P < 0.01, ***P < 0.001, and ****P < 0.0001. Comparison indications in Fig. 2C include # = *CD4-* or *LysM-cre* KO versus WT and ^ = *CD4-* or *LysM-cre* KO versus KO and Fig. 7 (H and I) include # = WT versus WT +DCA, * = WT versus KO, ^ = KO versus KO +DCA, and % = WT +DCA versus KO +DCA. Experiments and data analysis were not performed blinded to genotype. Data distribution for RNA-seq analyses and GSEA was assumed to be normal. All data were plotted and visualized with GraphPad Prism v10 or R packages clusterProfiler 4.11.0, ggplot2 3.5, or ComplexHeatmap 2.18.0 (*70*).

## Notes

**Correction (13 March 2026):** In the original article, there was a color error in the graph of Fig. 3E, where the top line was meant to be black and the lower line was meant to be pink. These colors have been corrected in the new version of Fig. 3. The authors’ results and conclusions are not affected by this change.

## Supplementary Materials

### The PDF file includes:

Figs. S1 to S6 Table S1 Legends for datasets S1, S2, S5, and S6 Datasets S3 and S4 References

### Other Supplementary Material for this manuscript includes the following:

Datasets S1, S2, S5, and S6
