# Supplementary material for: Mitochondrial dysfunction enhances influenza pathogenesis by up-regulating de novo sialic acid biosynthesis
Source: Sci Adv. 2025 Jul 4;11(27):eadu3739. doi: 10.1126/sciadv.adu3739 (PMC12227048; doi:10.1126/sciadv.adu3739)
Supplement: Supplementary file 1 — Figs. S1 to S6 Table S1 Legends for datasets S1, S2, S5, and S6 Datasets S3 and S4 References [file sciadv.adu3739_sm.pdf]

Science Advances
AAAS

Supplementary Materials for

Mitochondrial dysfunction enhances influenza pathogenesis by up-regulating de novo sialic acid biosynthesis

Amanda L. Fuchs *et al.*

Corresponding author: Peter J. McGuire, peter.mcguire@nih.gov

Sci. Adv. **11**, eadu3739 (2025)
DOI: 10.1126/sciadv.adu3739

The PDF file includes:

Figs. S1 to S6
Table S1
Legends for datasets S1, S2, S5, and S6
Datasets S3 and S4
References

Other Supplementary Material for this manuscript includes the following:

Datasets S1, S2, S5, and S6

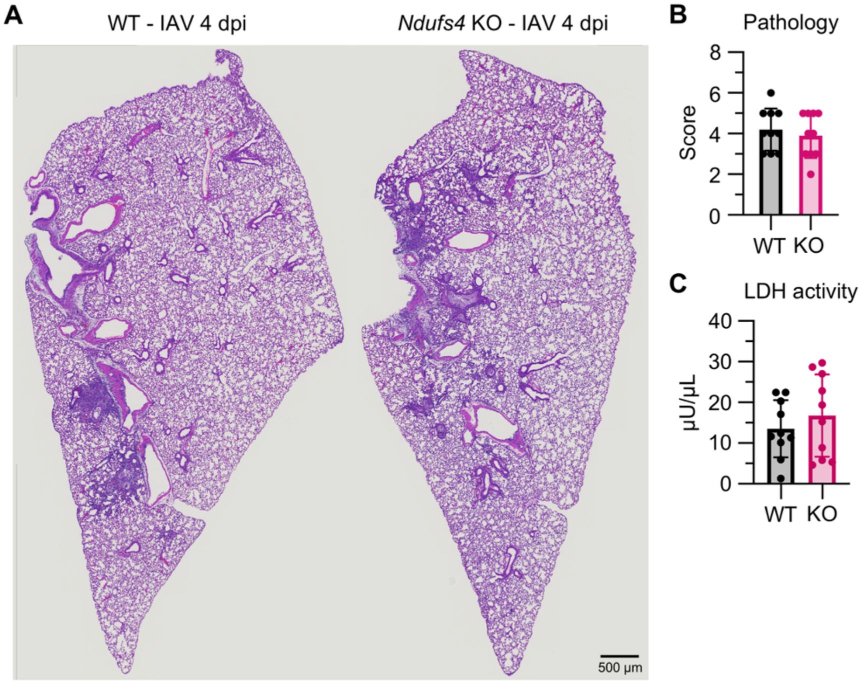

Fig. S1. IAV infection does not induce enhanced lung pathology in *Ndufs4* KO mice relative to wild type (WT) at 4 days post-infection (dpi). **(A)** Representative hematoxylin & eosin (H&E) images and **(B)** pathology scoring of WT and *Ndufs4* KO mouse left lung tissue sections at 4 dpi, n = 10 for WT and n = 10 for *Ndufs4* KO. Scale bar is 500 μm. **(C)** LDH activity levels in bronchoalveolar lavage (BAL) fluid collected from WT and *Ndufs4* KO mice at 4 dpi, n = 10 for WT and n = 10 for *Ndufs4* KO.

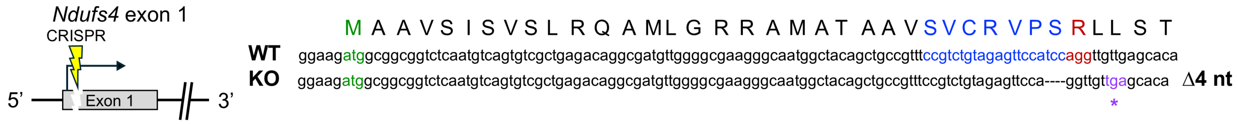

Fig. S2. CRISPR-mediated gene-targeting strategy to generate *Ndufs4* KO LET1 cells. Schematic drawing showing a portion of the *Ndufs4* exon 1 sequence in wild type (WT) and *Ndufs4* KO. Location of start codon (green), custom single guide RNA target sequence (blue), protospacer adjacent motif (PAM, red) and corresponding Ndufs4 protein sequence shown above. The CRISPR-induced double-stranded DNA break resulted in a frameshift mutation by deleting 4 nucleotides (nt) and thereby creation of a premature termination codon (*, purple).

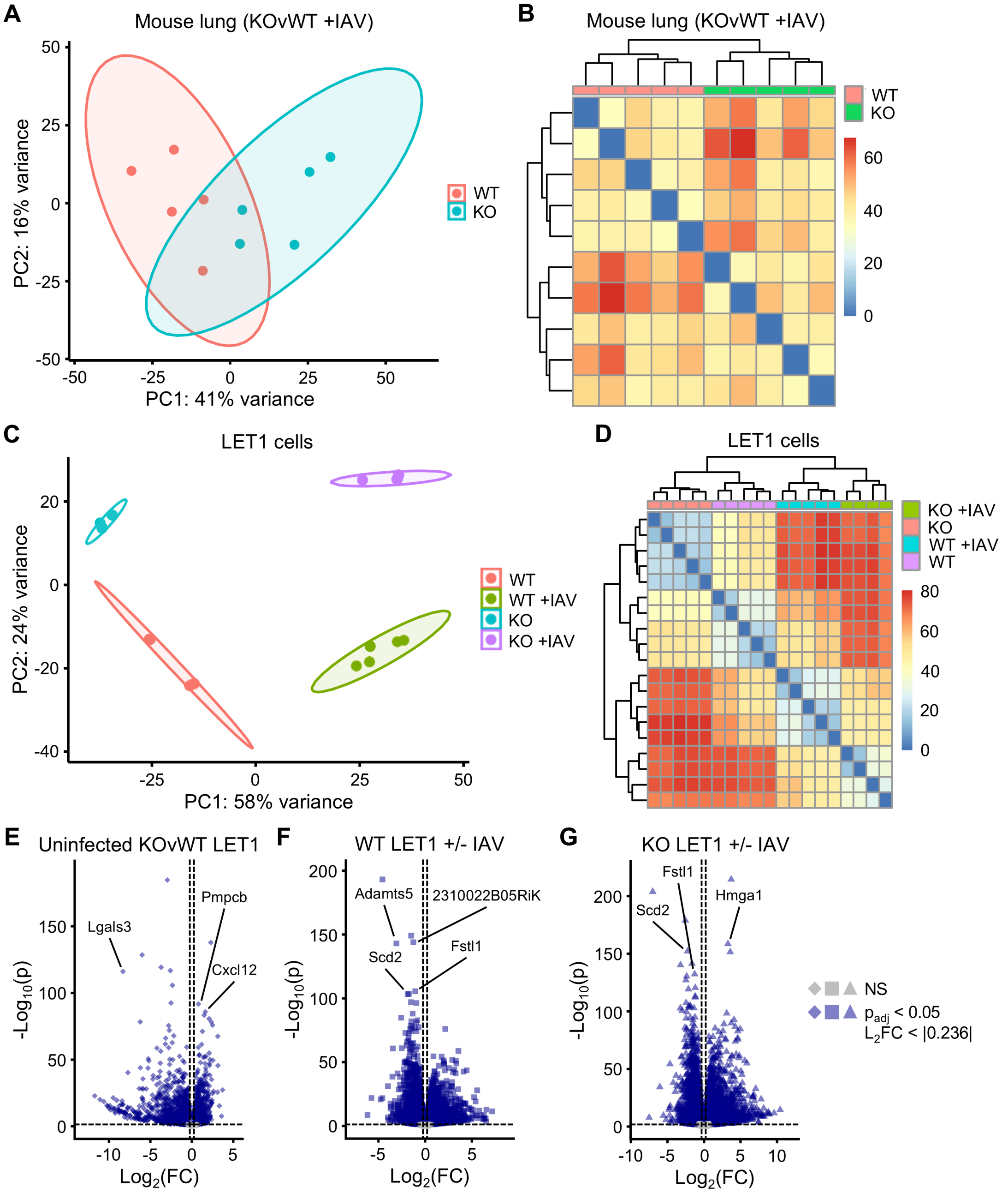

Fig. S3. Clustering analysis of RNA-seq datasets. (A) 2D principal component analysis (PCA) plot and **(B)** hierarchical clustering heatmap of IAV-infected wild type (WT) and *Ndufs4* KO mouse lung RNA-seq samples, n = 5 for WT and n = 5 for *Ndufs4* KO. **(C)** 2D PCA plot and **(D)** hierarchical clustering heatmap of uninfected and IAV-infected WT and *Ndufs4* KO LET1 RNA-seq samples, n ≥ 3 for WT, n = 5 for WT +IAV, n ≥ 3 for *Ndufs4* KO, and n ≥ 3 for *Ndufs4* KO +IAV. Volcano plots of differential expression analysis of **(E)** uninfected *Ndufs4* KO and WT LET1 (diamonds), **(F)** un- and IAV-infected WT LET1 (squares), and **(G)** un- and IAV-infected *Ndufs4* KO LET1 RNA-seq samples (triangles).

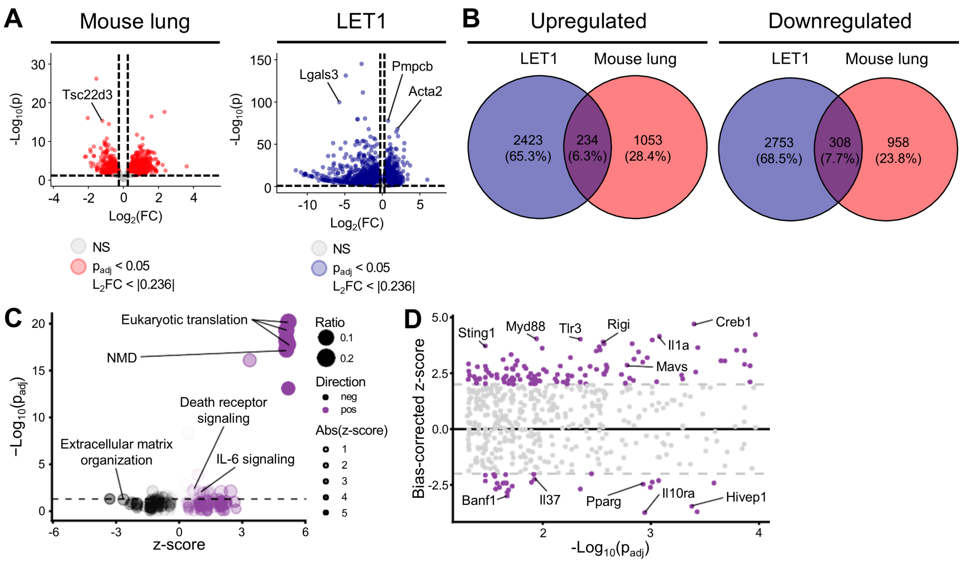

Fig. S4. Bioinformatic assessment of representative nature of the LET1 *Ndufs4* KO model. **(A)** Volcano plots of differential expression between IAV-infected *Ndufs4* KO and wild type (WT) mouse lung tissue (red) and LET1 cells (blue). **(B)** Venn diagrams of overlapping significantly upregulated and downregulated genes, from differential expression between IAV-infected *Ndufs4* KO and WT mouse lung tissue and LET1 cells. **(C)** Overrepresentation pathway analysis of intersecting genes. Bubble size reflects ratio of enriched genes to genes contained in the set, opacity reflects z-score, and color reflects directionality of enrichment. **(D)** Upstream regulator analysis of overrepresented upregulated and downregulated genes.

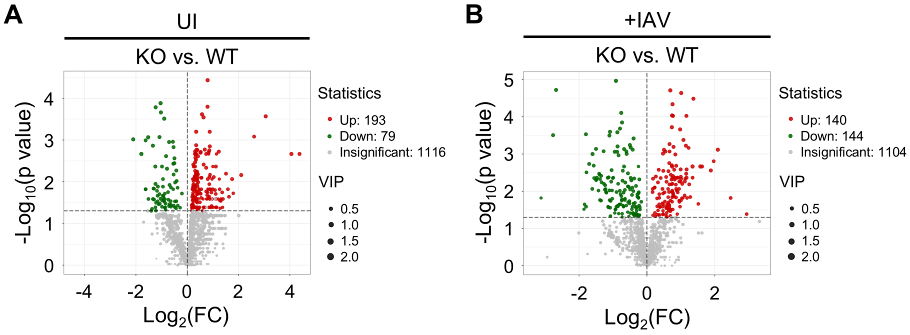

Fig. S5. Untargeted metabolomics analysis of *Ndufs4* KO and WT LET1 cells. Volcano plots of differential metabolites between **(A)** uninfected (UI) and **(B)** IAV-infected *Ndufs4* KO vs. wild type (WT) LET1 cells. Each point in the volcano plot represents a metabolite with green points representing down-regulation, red points representing up-regulation, and gray points representing detection but no significant difference. The x-axis represents fold change (FC) [$\log_2$(FC)] value of metabolites between groups, and the y-axis represents the significance level [$-\log_{10}$(p value)]. The size of each dot represents the variable importance in projection (VIP) value.

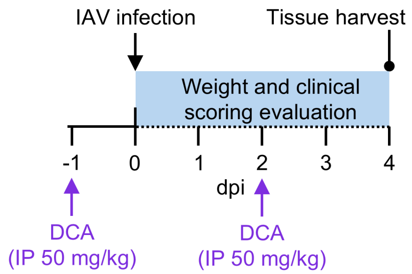

Fig. S6. Intraperitoneal (IP) 50 mg/kg DCA treatment regimen of P30 wild type (WT) and *Ndufs4* KO mice during IAV infection.

Table S1. Criteria for clinical severity scoring (CSS) in mice.*

	Score points	
**Body weight (BW) loss**		
– No change	0	
– Δ1-19%	1-19	
– Δ≥20%	20	
**General body condition**		
– Fur:		
– – Shiny	0	
– – Matte	2	
– – Ruffled	4	
– Eyes:		
– – Clean and clear	0	
– – Sticky	3	
– Posture:		
– – Normal	0	
– – Hunched	10	
– Complications:		
– – Cold to touch	20	
**Motility**		
– Normal	0	
– Reduced	1	
– Moderately reduced	2	
– Only when prodded	5	
– Isolation, lethargy	10	
**Respiration**		
– Normal	0	
– Slightly abnormal	1	
– Accelerated	10	
**CSS rating**	**Sum of score points**	**Description**
Severity level 0	0-3	None, healthy animals
Severity level 1	4-9	Low burden/illness, animal observation
Severity level 2	10-15	Moderate burden/illness, careful animal observation
Severity level 3	16-20	Moderate to severe burden/illness, careful animal observation
Severity level 4	>20	Severe burden/illness, animals moribund, humane endpoint

*adapted from S. Blättner et al. (*58*).

Data S1. (separate xlsx file)

Overrepresentation pathway analysis of intersecting upregulated and downregulated genes in IAV-infected LET1 and mouse lung data sets.

Data S2. (separate xlsx file)

Upstream regulator analysis of overrepresented upregulated and downregulated genes in IAV-infected LET1 and mouse lung data sets.

Data S3. X31 gene segment viral reads (scaled to gene) in IAV-infected *Ndufs4* KO and WT mouse lung tissues.

**Gene Segment**	A1WT	A2WT	A3WT	A4WT	A5WT	B1KO	B2KO	B3KO	B4KO	B5KO
X31_PB2	1.905	1.457	0.490	0.745	0.403	3.282	1.624	1.073	0.899	1.152
X31_PB1	2.338	0.980	0.829	0.476	0.375	2.978	2.500	1.405	2.228	2.261
X31_PA	2.108	0.972	0.603	0.790	0.527	2.730	2.170	1.544	1.530	2.034
X31_HA	2.359	0.943	0.611	0.652	0.435	3.414	2.486	3.146	2.121	1.504
X31_NP	1.677	1.189	0.802	0.655	0.677	2.099	1.512	1.877	1.319	1.498
X31_NA	1.876	1.101	0.494	0.990	0.539	2.298	1.628	2.179	1.333	1.486
X31_M	2.073	1.220	0.672	0.582	0.453	3.239	2.045	1.807	1.387	1.654
X31_NS	2.026	1.289	0.677	0.545	0.463	4.299	1.881	2.383	1.917	1.781

Data S4. X31 gene segment viral reads (scaled to batch and gene) in IAV-infected *Ndufs4* KO and WT LET1 cells.

**Gene Segment**	B1WTI1	B2WTI2	B3WTI3	G1WTI4	G2WTI5	D2KOI2	D3KOI3	H1KOI4	H2KOI5
X31_PB2	0.980	1.095	0.925	1.272	0.728	10.575	5.583	2.629	3.899
X31_PB1	0.978	1.078	0.944	1.301	0.699	11.021	6.496	2.029	3.351
X31_PA	0.994	1.078	0.928	1.294	0.706	8.901	5.248	2.005	3.275
X31_HA	1.013	1.028	0.958	1.323	0.677	6.335	3.494	1.960	3.258
X31_NP	0.945	1.023	1.032	1.244	0.756	2.984	2.055	1.270	1.726
X31_NA	0.985	1.066	0.948	1.312	0.688	8.115	4.310	2.031	3.159
X31_M	0.997	1.074	0.929	1.304	0.696	7.279	4.260	2.032	3.350
X31_NS	0.999	1.076	0.926	1.272	0.728	6.880	3.774	2.142	3.404

Data S5. (separate xlsx file)
Differential metabolites in uninfected *Ndufs4* KO vs. WT LET1 cells.

Data S6. (separate xlsx file)
Differential metabolites in IAV-infected *Ndufs4* KO vs. WT LET1 cells.

REFERENCES AND NOTES

1. A. B. Fisher, Intermediary metabolism of the lung. *Environ. Health Perspect.* **55**, 149–158 (1984).

2. R. F. Hoffmann, M. R. Jonker, S. M. Brandenburg, H. G. de Bruin, N. H. T. Ten Hacken, A. J. M. van Oosterhout, I. H. Heijink, Mitochondrial dysfunction increases pro-inflammatory cytokine production and impairs repair and corticosteroid responsiveness in lung epithelium. *Sci. Rep.* **9**, 15047 (2019).

3. J. Katzen, M. F. Beers, Contributions of alveolar epithelial cell quality control to pulmonary fibrosis. *J. Clin. Invest.* **130**, 5088–5099 (2020).

4. S. Garcia, A. Saldana-Caboverde, M. Anwar, A. P. Raval, N. Nissanka, M. Pinto, C. T. Moraes, F. Diaz, Enhanced glycolysis and GSK3 inactivation promote brain metabolic adaptations following neuronal mitochondrial stress. *Hum. Mol. Genet.* **31**, 692–704 (2022).

5. L. Ren, W. Zhang, J. Zhang, J. Zhang, H. Zhang, Y. Zhu, X. Meng, Z. Yi, R. Wang, Influenza A virus (H1N1) infection induces glycolysis to facilitate viral replication. *Virol. Sin.* **36**, 1532–1542 (2021).

6. J. B. Ritter, A. S. Wahl, S. Freund, Y. Genzel, U. Reichl, Metabolic effects of influenza virus infection in cultured animal cells: Intra- and extracellular metabolite profiling. *BMC Syst. Biol.* **4**, 61 (2010).

7. H. S. Smallwood, S. Duan, M. Morfouace, S. Rezinciuc, B. L. Shulkin, A. Shelat, E. E. Zink, S. Milasta, R. Bajracharya, A. J. Oluwaseum, M. F. Roussel, D. R. Green, L. Pasa-Tolic, P. G. Thomas, Targeting metabolic reprogramming by influenza infection for therapeutic intervention. *Cell Rep.* **19**, 1640–1653 (2017).

8. G. Haji, C. H. Wiegman, C. Michaeloudes, M. S. Patel, K. Curtis, P. Bhavsar, M. I. Polkey, I. M. Adcock, K. F. Chung, COPDMAP consortium, Mitochondrial dysfunction in airways and quadriceps muscle of patients with chronic obstructive pulmonary disease. *Respir. Res.* **21**, 262 (2020).

9. F. Sanei, T. Wilkinson, Influenza vaccination for patients with chronic obstructive pulmonary disease: Understanding immunogenicity, efficacy and effectiveness. *Ther. Adv. Respir. Dis.* **10**, 349–367 (2016).

10. T. N. Tarasenko, S. E. Pacheco, M. K. Koenig, J. Gomez-Rodriguez, S. M. Kapnick, F. Diaz, P. M. Zerfas, E. Barca, J. Sudderth, R. J. DeBerardinis, R. Covian, R. S. Balaban, S. DiMauro, P. J. McGuire, Cytochrome c oxidase activity is a metabolic checkpoint that regulates cell fate decisions during T cell activation and differentiation. *Cell Metab.* **25**, 1254–1268.e7 (2017).

11. S. E. Kruse, W. C. Watt, D. J. Marcinek, R. P. Kapur, K. A. Schenkman, R. D. Palmiter, Mice with mitochondrial complex I deficiency develop a fatal encephalomyopathy. *Cell Metab.* **7**, 312–320 (2008).

12. M. A. Ray, N. A. Johnston, S. Verhulst, R. A. Trammell, L. A. Toth, Identification of markers for imminent death in mice used in longevity and aging research. *J. Am. Assoc. Lab. Anim. Sci.* **49**, 282–288 (2010).

13. R. A. Trammell, L. A. Toth, Markers for predicting death as an outcome for mice used in infectious disease research. *Comp. Med.* **61**, 492–498 (2011).

14. D. Verhoeven, J. R. Teijaro, D. L. Farber, Pulse-oximetry accurately predicts lung pathology and the immune response during influenza infection. *Virology* **390**, 151–156 (2009).

15. H. T. Groves, J. U. McDonald, P. Langat, E. Kinnear, P. Kellam, J. McCauley, J. Ellis, C. Thompson, R. Elderfield, L. Parker, W. Barclay, J. S. Tregoning, Mouse models of influenza infection with circulating strains to test seasonal vaccine efficacy. *Front. Immunol.* **9**, 126 (2018).

16. Z. Jin, W. Wei, M. Yang, Y. Du, Y. Wan, Mitochondrial complex I activity suppresses inflammation and enhances bone resorption by shifting macrophage-osteoclast polarization. *Cell Metab.* **20**, 483–498 (2014).

17. M. Jestin, S. M. Kapnick, T. N. Tarasenko, C. T. Burke, P. M. Zerfas, F. Diaz, H. Vernon, L. N. Singh, R. J. Sokol, P. J. McGuire, Mitochondrial disease disrupts hepatic allostasis and lowers the threshold for immune-mediated liver toxicity. *Mol. Metab.* **37**, 100981 (2020).

18. S. M. Cloonan, A. M. K. Choi, Mitochondria in lung disease. *J. Clin. Invest.* **126**, 809–820 (2016).

19. C. M. Rosenberger, R. L. Podyminogin, P. S. Askovich, G. Navarro, S. M. Kaiser, C. J. Sanders, J. L. McClaren, V. C. Tam, P. Dash, J. G. Noonan, B. G. Jones, S. L. Surman, J. J. Peschon, A. H. Diercks, J. L. Hurwitz, P. C. Doherty, P. G. Thomas, A. Aderem, Characterization of innate responses to influenza virus infection in a novel lung type I epithelial cell model. *J. Gen. Virol.* **95**, 350–362 (2014).

20. A. Ames III, Energy requirements of CNS cells as related to their function and to their vulnerability to ischemia: A commentary based on studies on retina. *Can. J. Physiol. Pharmacol.* **70**, S158–S164 (1992).

21. L. Da Dalt, A. G. Cabodevilla, I. J. Goldberg, G. D. Norata, Cardiac lipid metabolism, mitochondrial function, and heart failure. *Cardiovasc. Res.* **119**, 1905–1914 (2023).

22. S. M. Ostojic, Tackling guanidinoacetic acid for advanced cellular bioenergetics. *Nutrition* **34**, 55–57 (2017).

23. E. Trefts, A. S. Williams, D. H. Wasserman, Exercise and the regulation of hepatic metabolism. *Prog. Mol. Biol. Transl. Sci.* **135**, 203–225 (2015).

24. A. J. Benie, A. Blume, R. R. Schmidt, W. Reutter, S. Hinderlich, T. Peters, Characterization of ligand binding to the bifunctional key enzyme in the sialic acid biosynthesis by NMR: II. Investigation of the ManNAc kinase functionality. *J. Biol. Chem.* **279**, 55722–55727 (2004).

25. A. Paneque, H. Fortus, J. Zheng, G. Werlen, E. Jacinto, The hexosamine biosynthesis pathway: Regulation and function. *Genes* **14**, 933 (2023).

26. S. G. Sampathkumar, A. V. Li, K. J. Yarema, Synthesis of non-natural ManNAc analogs for the expression of thiols on cell-surface sialic acids. *Nat. Protoc.* **1**, 2377–2385 (2006).

27. N. K. Sauter, J. E. Hanson, G. D. Glick, J. H. Brown, R. L. Crowther, S. J. Park, J. J. Skehel, D. C. Wiley, Binding of influenza virus hemagglutinin to analogs of its cell-surface receptor, sialic acid: Analysis by proton nuclear magnetic resonance spectroscopy and X-ray crystallography. *Biochemistry* **31**, 9609–9621 (1992).

28. S. Eguchi, N. Oshiro, T. Miyamoto, K. Yoshino, S. Okamoto, T. Ono, U. Kikkawa, K. Yonezawa, AMP-activated protein kinase phosphorylates glutamine: Fructose-6-phosphate amidotransferase 1 at Ser243 to modulate its enzymatic activity. *Genes Cells* **14**, 179–189 (2009).

29. S. Kirkeby, C. J. Martel, B. Aasted, Infection with human H1N1 influenza virus affects the expression of sialic acids of metaplastic mucous cells in the ferret airways. *Virus Res.* **144**, 225–232 (2009).

30. T. J. Carroll, B. Caraet, N. Madsen, D. Wilbur, Development of de Quervain tenosynovitis after distal radius fracture. *Hand* **19**, 1154–1158 (2024).

31. A. Sugunan, K. Rajasekharan Pillai, A. George, Effectiveness of interventions to contain out-of-pocket-expenditure in lower-middle income countries: A systematic review and synthesis. *Int. J. Health Plann. Manage.* **38**, 918–935 (2023).

32. N. Giovannone, A. Antonopoulos, J. Liang, J. Geddes Sweeney, M. R. Kudelka, S. L. King, G. S. Lee, R. D. Cummings, A. Dell, S. R. Barthel, H. R. Widlund, S. M. Haslam, C. J. Dimitroff, Human B cell differentiation is characterized by progressive remodeling of O-linked glycans. *Front. Immunol.* **9**, 2857 (2018).

33. S. Zhang, Y. Wu, Z. Xuan, X. Chen, J. Zhang, D. Ge, X. Wang, Screening differential miRNAs responsible for permeability increase in HUVECs infected with influenza A virus. *PLOS ONE* **12**, e0186477 (2017).

34. N. Hahon, J. A. Booth, H. L. Eckert, Cell attachment and penetration by influenza virus. *Infect. Immun.* **7**, 341–351 (1973).

35. T. P. Sutula, N. B. Fountain, 2DG and glycolysis as therapeutic targets for status epilepticus. *Epilepsy Behav.* **140**, 109108 (2023).

36. C. L. R. van Doorn, G. K. Schouten, S. van Veen, K. V. Walburg, J. J. Esselink, M. T. Heemskerk, F. Vrieling, T. H. M. Ottenhoff, Pyruvate dehydrogenase kinase inhibitor dichloroacetate improves host control of Salmonella enterica serovar typhimurium infection in human macrophages. *Front. Immunol.* **12**, 739938 (2021).

37. J. Bennett, M. Kerr, S. C. Greenway, M. W. Friederich, J. L. K. Van Hove, D. Hittel, A. Khan, Improved lactate control with dichloroacetate in a case with severe neonatal lactic acidosis due to MTFMT mitochondrial translation disorder. *Mol. Genet. Metab. Rep.* **24**, 100616 (2020).

38. A. W. El-Hattab, A. M. Zarante, M. Almannai, F. Scaglia, Therapies for mitochondrial diseases and current clinical trials. *Mol. Genet. Metab.* **122**, 1–9 (2017).

39. S. M. Kapnick, S. E. Pacheco, P. J. McGuire, The emerging role of immune dysfunction in mitochondrial diseases as a paradigm for understanding immunometabolism. *Metabolism* **81**, 97–112 (2018).

40. D. A. Alexander, K. Dimock, Sialic acid functions in enterovirus 70 binding and infection. *J. Virol.* **76**, 11265–11272 (2002).

41. N. Arnberg, A. H. Kidd, K. Edlund, J. Nilsson, P. Pring-Akerblom, G. Wadell, Adenovirus type 37 binds to cell surface sialic acid through a charge-dependent interaction. *Virology* **302**, 33–43 (2002).

42. L. D. Cahan, J. C. Paulson, Polyoma virus adsorbs to specific sialyloligosaccharide receptors on erythrocytes. *Virology* **103**, 505–509 (1980).

43. M. Matrosovich, G. Herrler, H. D. Klenk, Sialic acid receptors of viruses. *Top. Curr. Chem.* **367**, 1–28 (2015).

44. M. R. Nokhbeh, S. Hazra, D. A. Alexander, A. Khan, M. McAllister, E. J. Suuronen, M. Griffith, K. Dimock, Enterovirus 70 binds to different glycoconjugates containing α2,3-linked sialic acid on different cell lines. *J. Virol.* **79**, 7087–7094 (2005).

45. J. J. Skehel, D. C. Wiley, Receptor binding and membrane fusion in virus entry: The influenza hemagglutinin. *Annu. Rev. Biochem.* **69**, 531–569 (2000).

46. R. Vlasak, W. Luytjes, W. Spaan, P. Palese, Human and bovine coronaviruses recognize sialic acid-containing receptors similar to those of influenza C viruses. *Proc. Natl. Acad. Sci. U.S.A.* **85**, 4526–4529 (1988).

47. M. C. Gingras, A. Pause, FLCN: A new regulator of AMPK-dependent Warburg metabolic reprogramming. *Mol. Cell. Oncol.* **1**, e961819 (2014).

48. S. C. Johnson, M. E. Yanos, E. B. Kayser, A. Quintana, M. Sangesland, A. Castanza, L. Uhde, J. Hui, V. Z. Wall, A. Gagnidze, K. Oh, B. M. Wasko, F. J. Ramos, R. D. Palmiter, P. S. Rabinovitch, P. G. Morgan, M. M. Sedensky, M. Kaeberlein, mTOR inhibition alleviates mitochondrial disease in a mouse model of Leigh syndrome. *Science* **342**, 1524–1528 (2013).

49. M. Martin-Perez, A. S. Grillo, T. K. Ito, A. S. Valente, J. Han, S. W. Entwisle, H. Z. Huang, D. Kim, M. Yajima, M. Kaeberlein, J. Villén, PKC downregulation upon rapamycin treatment attenuates mitochondrial disease. *Nat. Metab.* **2**, 1472–1481 (2020).

50. C. Ehrhardt, T. Wolff, S. Pleschka, O. Planz, W. Beermann, J. G. Bode, M. Schmolke, S. Ludwig, Influenza A virus NS1 protein activates the PI3K/Akt pathway to mediate antiapoptotic signaling responses. *J. Virol.* **81**, 3058–3067 (2007).

51. Y. K. Shin, Q. Liu, S. K. Tikoo, L. A. Babiuk, Y. Zhou, Influenza A virus NS1 protein activates the phosphatidylinositol 3-kinase (PI3K)/Akt pathway by direct interaction with the p85 subunit of PI3K. *J. Gen. Virol.* **88**, 13–18 (2007).

52. M. Keshavarz, F. Solaymani-Mohammadi, H. Namdari, Y. Arjeini, M. J. Mousavi, F. Rezaei, Metabolic host response and therapeutic approaches to influenza infection. *Cell. Mol. Biol. Lett.* **25**, 15 (2020).

53. E. L. Mazo, V. A. Rusiaev, A. N. Fedorov, N. G. Iaroslavtseva, I. G. Kharitonenkov, [Effect of the matrix protein of the influenza virus on oxidative phosphorylation in preparations of isolated liver mitochondria from white mice]. *Vopr. Virusol.* **33**, 153–157 (1988).

54. B. A. Barshop, R. K. Naviaux, K. A. McGowan, F. Levine, W. L. Nyhan, A. Loupis-Geller, R. H. Haas, Chronic treatment of mitochondrial disease patients with dichloroacetate. *Mol. Genet. Metab.* **83**, 138–149 (2004).

55. M. Lavorato, E. Nakamaru-Ogiso, N. D. Mathew, E. Herman, N. Shah, S. Haroon, R. Xiao, C. Seiler, M. J. Falk, Dichloroacetate improves mitochondrial function, physiology, and morphology in FBXL4 disease models. *JCI Insight* **7**, e156346 (2022).

56. A. R. Hanaford, A. Khanna, K. James, V. Truong, R. Liao, Y. Chen, M. Mulholland, E. B. Kayser, K. Watanabe, E. S. Hsieh, M. Sedensky, P. G. Morgan, V. Kalia, S. Sarkar, S. C. Johnson, Interferon-gamma contributes to disease progression in the *Ndufs4*(−/−) model of Leigh syndrome. *Neuropathol. Appl. Neurobiol.* **50**, e12977 (2024).

57. J. C. Stokes, R. L. Bornstein, K. James, K. Y. Park, K. A. Spencer, K. Vo, J. C. Snell, B. M. Johnson, P. G. Morgan, M. M. Sedensky, N. A. Baertsch, S. C. Johnson, Leukocytes mediate disease pathogenesis in the *Ndufs4*(KO) mouse model of Leigh syndrome. *JCI Insight* **7**, e156522 (2022).

58. S. Blättner, S. Das, K. Paprotka, U. Eilers, M. Krischke, D. Kretschmer, C. W. Remmele, M. Dittrich, T. Müller, C. Schuelein-Voelk, T. Hertlein, M. J. Mueller, B. Huettel, R. Reinhardt, K. Ohlsen, T. Rudel, M. J. Fraunholz, Staphylococcus aureus exploits a non-ribosomal cyclic dipeptide to modulate survival within epithelial cells and phagocytes. *PLOS Pathog.* **12**, e1005857 (2016).

59. A. F. Payne, I. Binduga-Gajewska, E. B. Kauffman, L. D. Kramer, Quantitation of flaviviruses by fluorescent focus assay. *J. Virol. Methods* **134**, 183–189 (2006).

60. M. I. Love, W. Huber, S. Anders, Moderated estimation of fold change and dispersion for RNA-seq data with DESeq2. *Genome Biol.* **15**, 550 (2014).

61. A. Zhu, J. G. Ibrahim, M. I. Love, Heavy-tailed prior distributions for sequence count data: Removing the noise and preserving large differences. *Bioinformatics* **35**, 2084–2092 (2019).

62. R. Patro, G. Duggal, M. I. Love, R. A. Irizarry, C. Kingsford, Salmon provides fast and bias-aware quantification of transcript expression. *Nat. Methods* **14**, 417–419 (2017).

63. G. M. Boratyn, J. Thierry-Mieg, D. Thierry-Mieg, B. Busby, T. L. Madden, Magic-BLAST, an accurate RNA-seq aligner for long and short reads. *BMC Bioinformatics* **20**, 405 (2019).

64. T. Wu, E. Hu, S. Xu, M. Chen, P. Guo, Z. Dai, T. Feng, L. Zhou, W. Tang, L. Zhan, X. Fu, S. Liu, X. Bo, G. Yu, clusterProfiler 4.0: A universal enrichment tool for interpreting omics data. *Innovation* **2**, 100141 (2021).

65. L. Krenacs, T. Krenacs, E. Stelkovics, M. Raffeld, Heat-induced antigen retrieval for immunohistochemical reactions in routinely processed paraffin sections. *Methods Mol. Biol.* **588**, 103–119 (2010).

66. B. Kimble, G. R. Nieto, D. R. Perez, Characterization of influenza virus sialic acid receptors in minor poultry species. *Virol. J.* **7**, 365 (2010).

67. H. Nakano, K. Nakano, D. N. Cook, Isolation and purification of epithelial and endothelial cells from mouse lung. *Methods Mol. Biol.* **1799**, 59–69 (2018).

68. P. Pennitz, C. Goekeri, J. Trimpert, E. Wyler, A. Ebenig, C. Weissfuss, M. D. Mühlebach, M. Witzenrath, G. Nouailles, Protocol to dissociate healthy and infected murine- and hamster-derived lung tissue for single-cell transcriptome analysis. *STAR Protoc.* **4**, 101957 (2023).

69. P. M. Guire, J. Marin, A. Fuchs, T. Tarasenko, E. Warren, M. Kirby, S. Anderson, E. Gordon-Lipkin, S. Kruk, A. West, Mapping metabolic dependences and capacities using ATP as a biomarker (2025); https://doi.org/10.21203/rs.3.rs-4836421/v2.

70. Z. Gu, R. Eils, M. Schlesner, Complex heatmaps reveal patterns and correlations in multidimensional genomic data. *Bioinformatics* **32**, 2847–2849 (2016).
